# 5′,8-cyclo-dAdo and 8-oxo-dAdo DNA Lesions Are Both Substrates of Adenosine Deaminase: A Preliminary Study

**DOI:** 10.3390/cells14211665

**Published:** 2025-10-23

**Authors:** Bolesław T. Karwowski

**Affiliations:** Nucleic Acids Damage Laboratory, Faculty of Pharmacy, Medical University of Lodz, Ul. Muszynskiego 1, 90-151 Lodz, Poland; boleslaw.karwowski@umed.lodz.pl; Tel.: +48-722-220-711

**Keywords:** DNA damage, (5′*R*) and (5′*S*) 5′,8-cyclo-2′-deoxyadenosine, 7,8-dihydro-8-oxo-2′-deoxyadenosine, adenosine deaminase, density-functional tight-binding, high resolution mass spectroscopy, reverse-phase high-performance liquid chromatography

## Abstract

Genetic information, whether inside or outside the nucleus, is exposed to a variety of harmful physico-chemical factors. Although DNA damage repair systems have been extensively studied, little information about post-repair and non-genomic DNA damage metabolism is available in the literature. Adenosine deaminase (ADA) is an abundant enzyme found on both sides of the cell membrane that regulates the concentration of adenine derivatives. In this article, it has been shown that 7,8-dihydro-8-oxo-2′-deoxyadenosine (^OXO^dAdo) and (5′*R*/*S*) 5′,8-cyclo-2′-deoxyadenosine ((5′*R*/*S*)cdAdo) are suitable substrates for ADA. For this purpose, theoretical Density Functional Tight Binding and RP-HPLC analyses were applied. The products of ADA activity, i.e., ^OXO^dIno (7,8-dihydro-8-oxo-2′-deoxyinosine) and (5′*R*/*S*) cdIno ((5′*R*/*S*) 8-cyclo-2′-deoxyinosine), were identified and confirmed by high-resolution mass spectroscopy. Although the (5′*R*) and (5′*S*)cdAdo enzymatic deamination processes are much slower (34% and 32% after 168 h, respectively) than the process observed for dAdo, 5′,8-cyclo-2′-deoxyinosine should be considered when monitoring cyclopurine levels in physiological fluids. The same should be considered in the case of ^OXO^dAdo, which is completely converted to ^OXO^dIno within one minute and may therefore be less visible than ^OXO^dGuo during mass spectroscopy analysis. Both these observations are important, given the abundance of 2′-deoxyadenosine on both sides of the cell membrane and its potential conversion into ^OXO^dAdo and (5′*R*/*S*)cdAdo. They may also explain why the observed level of ^OXO^dAdo is much lower than that of ^OXO^dGuo in cells and physiological fluids, even though their difference in ionisation potential is only 0.25 eV. Future studies are needed to further investigate the metabolism of DNA damage and to identify the enzymes involved in nucleic acid biochemistry.

## 1. Introduction

The human genome contains 3.3 × 10^9^ nucleoside pairs, which exist in the cell nucleus as highly concentrated chromatin. This ‘seed of life’ is continuously exposed to various harmful intra- and extracellular chemical and physical factors, among which the hydroxyl radical (●OH), a reactive oxygen species (ROS), is the most reactive [1,2]. ●OH can be generated not only by the Haber–Weiss reaction, in which it is catalysed by transition metal ions, but also by ionising radiation, e.g., gamma radiolysis of water [3,4]. Although *A* hydroxyl radical has a relatively short lifetime (10^−10^ s), it can react with a wide variety of molecules with diffusion control (10^9^–10^10^ M^−1^ s^−1^) [5,6]. Its activity with nucleosides/nucleotides can lead to the abstraction of hydrogen atoms from the sugar moiety or their addition to the unsaturated bonds of nucleobases. This process can cause DNA damage, resulting in increased mutagenic potential in the genome if not correctly repaired [7]. Over the course of evolution, cells have developed many different repair mechanisms to protect genetic information, including DNA mismatch repair (MMR), long (LP-) and short patch (SP-) base excision repair (BER), nucleotide excision repair (NER) with two sub pathways: global genome (GG-) and transcription-coupled (TC-), homologous recombination (HR), classical non-homologous end joining (C-NHEJ) and “alternative” end joining (A-EJ) to protect genetic information [8,9].

Free nucleosides and nucleotides are not only part of DNA but are also moieties of RNA, messengers such as cyclic adenosine monophosphate (cAMP), and sources of energy such as adenosine triphosphate (ATP). Additionally, nucleosides are continuously synthesised in cells de novo, recycled by salvage pathways, or absorbed from the extracellular environment through special channels (transporters), such as hCNT1-3 or hENT1-4 [10,11]. Their abundance outside the genome implies that their probability of interacting with hydroxyl radicals or other ROS is higher than within the genome. To protect this seed of life against the incorporation of noncanonical nucleotides, specialised defence systems have evolved. The GO system, for example, which is based on glycosylase activity, removes 7,8-dihydro-8-oxo-2′-deoxyguanosine (^OXO^dGuo) from its pool by glycosidic bond hydrolysis or steric hindrance in the polymerase catalytic pocket/centre [12,13].

**Figure 1:**
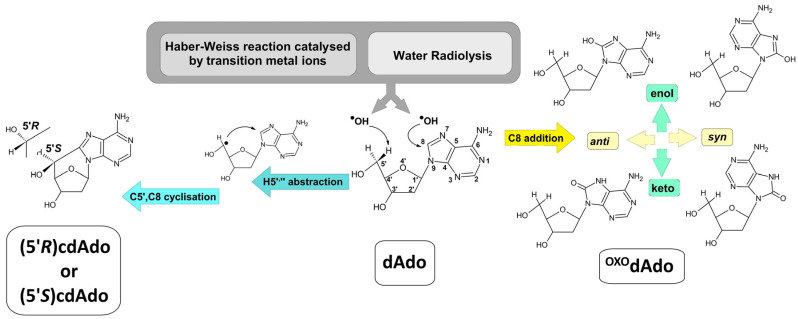
Graphical representation of cdAdo and ^OXO^dAdo formation after hydroxyl radical activity as a product of the Haber–Weiss reaction catalysed by transition metal ions or water radiolysis. The C5′,C8 endocyclisation initiated by hydrogen atom abstraction from dAdo cause to two diastereomers (5′*R*) and (5′*S*)cdAdo generation. The addition of ●OH to the C8 of dAdo leads to two conformers *syn* and *anti* of ^OXO^dAdo yield which can exist in enol and keto forms.

Nucleosides/nucleotides are also naturally present in the intercellular space as a result of food intake [14]. It was recently discovered that they can also be incorporated into genomic DNA as a product of hydroxyl radical activity, e.g., ^OXO^dGuo, thymine glycol [15]. Surprisingly, the cellular pool of lesioned nucleosides is powered by DNA repair machinery, such as NER or long-patch BER, which release mainly 20-mer/12-mer fragments, respectively, of excised nucleobases into the cytosol. These short DNA fragments are subsequently degraded by exo/endonucleases and phosphatases into their nucleosidic form, which can serve as a suitable substrate for cellular secretion or conversion to uric acid by adenosine/guanosine deaminase and xanthine oxidase [16,17]. Most canonical and non-canonical nucleosides, as well as DNA lesions, are suitable substrates for the above process. However, both diastereomers, i.e., (5′*R*) and (5′*S*) of cdAdo, are perceived as resistant to further conversion. 5′,8-cyclo-2′-deoxyadenosine ((5′*S*/*R*)cdAdo) is a tandem lesion whose formation is initiated by abstraction of the 5′ or 5” hydrogen atom by a hydroxyl radical with a subsequent C5′,C8 cyclisation reaction (Figure 1) [18]. The commonly accepted level of (5′*R*/*S*)cdAdo in the genome has been estimated to be 0.13/0.48 per 10^7^ 2′-deoxynucleosides (in mouse liver DNA) [19]. In comparison, the abundance of more common lesions, i.e., ^OXO^dAdo and ^OXO^dG, has been estimated as 2.30 ± 0.19 and 14.62 ± 1.45 per 10^6^ DNA bases, respectively. 5′,8-cyclopurines (cdPu) lesions are not a convenient substrate for currently known glycosylases; moreover, their presence in ds-DNA impedes the repair of other lesions by BER machinery [20,21].

Adenosine deaminase (ADA) is an enzyme present on both sides of the cell membrane. It converts adenosine derivatives, i.e., adenosine and 2′-deoxyadenine, to inosine and 2′-deoxyadenosine, respectively [22,23]. Additionally, this protein is able to inactivate the pharmacological compound cordycepin, which is, in fact, a noncanonical nucleoside [24]. ADA maintains the physiological homeostasis of ATP and other cellular messengers. ADA deficiency causes immunological problems, such as severe combined immunodeficiency (SCID) syndrome [25,26].

Notably, NER removes cdAdo from the genome much more slowly than *cis*-platin adducts, which makes it harmful for cells [27,28]. Even though the influence of cdPu on the stability of genetic information has been investigated by several groups over the last four decades, its cellular metabolism, following its release from DNA or formation as free nucleosides in intra- or extracellular environments, remains unknown. This research demonstrates that both diastereomers of cdAdo, 5′*R* and 5′*S,* and ^OXO^dAdo are suitable substrates for adenosine deaminase.

## 2. Materials and Methods

### 2.1. Materials

The starting materials/reagents were purchased from Merck (Poznan, Poland) and used directly without any further manipulation. The water was purified by a Milli-Q EQ 7000 Ultrapure Water Purification System before use; other solvents of analytical grade were used directly. The (5′*R*) and (5′*S*) 5′,8-cyclo-2′deoxyadenosine were synthesised as described previously by Romieu et al. [29,30]. ^OXO^dAdo was synthesised according to Navacia [31]. Adenosine deaminase (ADA), obtained from calf intestine (10 mg/2 mL; 200 U/mg, 1 U/μL), Roche Diagnostics GmbH, Mannheim, Germany (Ref 10102105001), was purchased from Merck (Poznan, Poland).

### 2.2. Spectroscopic Analysis

Nucleoside concentration was determined using a Varian Cary 1.3E spectrophotometer (Varian, Brunn am Gebirge, Austria). The maximum absorbance wavelength was measured as follows: ^OXO^dAdo = 270 nm; (5′*R*)cdAdo = 266 nm; (5′*S*)cdAdo = 265 nm.

High-resolution mass spectrometry (HR MS) measurements were performed using a Synapt G2-Si mass spectrometer (Waters Corp., Milford, MA, USA) equipped with an ESI source and a quadrupole-Time-of-Flight mass analyser, as described previously [32]. The mass spectrometer was used in the positive or negative ion detection mode. The optimised source parameters were as follows: 2.7 kV of capillary voltage; 30 V of cone voltage; 110 °C of source temperature; and nitrogen desolvation gas at 600 dm^3^/h, 350 °C, and 6.5 bar nebuliser gas. To ensure accurate mass measurements, data were collected in centroid mode. A leucine encephalin solution, Lock-Spray TM, (Waters Corp., Milford, MA, USA), was used as an external reference for mass correction during acquisition, which generated a reference ion at *m*/*z* 554.2615 ([M − H]^−^) in the negative ESI mode and at *m*/*z* 556.2771 Da ([M + H]^+^) in the positive ESI (electrospray ionisation) mode. Argon was used as a neutral gas for collision-induced fragmentation experiments. The collision energy was set at 20, 25 and 30 eV. The results of the measurements were processed using MassLynx 4.1 software (Waters).

### 2.3. General Procedure for Adenosine Deaminase Activity Assay by RP HPLC

The adenosine deaminase activity assay was performed in 0.5 mL of a reaction mixture comprising 50 mM HEPES (4-(2-hydroxyethyl)-1-piperazineethanesulfonic acid) buffer at a pH of 7.3. An appropriate amount (32.9 nM; 8.26 μg, corresponding to 0.5 [OD]) of the investigated nucleosides, i.e., (5′*R*)cdAde, (5′*S*)cdAde, and ^OXO^dAde, was added. The reaction was initiated by the addition of an appropriate amount of adenosine deaminase (275 μg; 55 μL; 55 U) to the nucleoside solution. The deamination process was monitored at 37 °C using RP-HPLC (reverse-phase high-performance liquid chromatography). HPLC spectra within a 190–400 nm detection range were archived every 24 h for 168 h. The abovementioned enzymatic experiments were conducted in triplicate.

Each RP-HPLC analysis was performed using a C-18 column, the Synergi 4 μm Fusion-RP 80 Å, 250 × 4.6 mm (Shimadzu, Kyoto, Japan), at a flow rate of 0.5 mL/min and with UV detection in the range of 190–400 nm. The following buffers were used: (A) 0.1 M ammonium acetate (pH 6.82 at 21 °C) and (B) CH_3_CN. The HPLC analytical gradient was as follows: from 0 to 25 min, the concentration of B increased from 0 to 10%, subsequently increased to 80% from 25 to 35 min, remained at this level for five minutes, and subsequently decreased to 0% within 10 min; the equilibration time was five minutes.

### 2.4. Geometry Optimisation and Assigment of Interaction Energy [32]

The initial geometries of (5′*R*) 5′,8-cyclo-2′-deoxyadenosine, (5′*S*) 5′,8-cyclo-2′-deoxyadenosine and *syn*/*anti* 7,8-dihydro-8-oxo-2′-deoxyadenosine were obtained by modifying the 2′-deoxycoformycin (Pentostatine) structure from its complex with adenosine deaminase (1a4l.pdb) [33]. The amino acids chains, crystal water moieties, and Zn^2+^ ion were left unmodified (as described previously). The spatial geometry of the Michaelis complex, i.e., ADA/*syn* ^OXO^dAdo, ADA/*anti* ^OXO^dAdo, ADA/(5′*R*)cdAdo, and ADA/(5′*S*)cdAdo, was optimised using SCC-DFTB methodology [34] (Self-Consistent redistribution of Mulliken Charge modification—Density Functional Tight Binding) [35] with the 3ob-3-1 parameter set was applied (Third-order parametrisation for organic and biological systems) [36]. Additionally, the Minnesota Solvation Model 12 (SM12) was used [37]. The generalised Born/solvent-accessible surface area model (GBSA) for water was applied, using a solvent-accessible angular surface grid of 230. The convergence criterion for the SCC-DFTB interaction was set at ≤10^−5^ Hartree.

The interaction energy (IE) in Kcal mol^−1^ was calculated at the same theoretical level as above, i.e., SCC-DFTB/3ob-3-1 in the aqueous phase (SM12). The interaction energy was defined as the difference between the Michaelis complex energy (*E_MC_*) and the sum of the energies of the isolated moieties, i.e., the adenosine deaminase moiety (*E_ADA_*) and adenosine derivatives (*E_ade der_.*): *IE* = *E_MC_* − (*E_ADA_* + *E_dAdo_*). The molecular ADF (Amsterdam Density Functional) program suite, version 2023.101 (part of Software for Chemistry & Materials B.V. Amsterdam, The Netherlands), was used for the theoretical geometry optimisations and energy calculations [38].

## 3. Results

**Figure 2:**
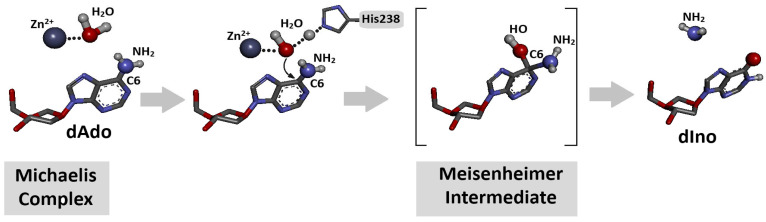
Schematic overview of conversion of 2′-deoxyadenosine to 2′-deoxyinosine (deamination) by adenosine deaminase (ADA) in the presence of H_2_O molecule and Zn^2+^ ion after Michaelis complex formation [32]. The process is initiated by direct nucleophilic attack of activated water molecule on C6 position of dAde with Meisenheimer (tetrahedral) intermediate creation. Initial spatial structure was isolated from previously discussed ADA-dAdo complex [32]. Atom colour: blue Nitrogen, Red Oxygen, dark grey Carbon, light grey Hydrogen.

Extra- and intracellular fluids are full of nucleosides and nucleotides, which are the building blocks of DNA and RNA and are continually exposed to harmful factors, such as hydroxyl radicals or to one-electron oxidation processes. Among the canonical nucleobases, guanine has the lowest ionisation potential, followed by adenine. Both purine nucleosides are susceptible to C8 oxidation, which, in the case of dAdo, leads to ^OXO^dAdo formation. Conversely, (5′*R*/*S*)cdAdo is yielded exclusively by hydroxyl radical activity, as shown in Figure 2 [39,40]. As both lesioned nucleosides are derivatives of adenine, they are potentially suitable substrates for ADA. However, this possibility has never been explored. To examine this phenomenon, the ADA activity on (5′*R*/*S*)cdAdo and ^OXO^dAdo was investigated under RP-HPLC control (monitoring).

### 3.1. PR-HPLC Studies of (5′R/S)cdA and ^OXO^dAdo Conversion to (5′R/S)cdIno and ^OXO^dIno by Adenine Deaminase

Adenine and its nucleoside derivatives, adenosine and 2′-deoxyadenosine, are suitable substrates for adenosine deaminase, which belongs to the aminohydrolase enzyme group. The mechanistic details of the deamination process were described in Section 3.3 and are shown in Figure 2 above [33].

**Figure 3:**
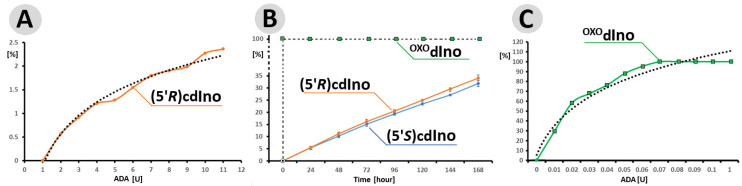
(**A**) Efficiency of (5′*R*)cdAdo (0.1 [OD]) deamination by ADA in the range 1–12 [U] after 24 h at 37 °C. (**B**) Profile of (5′*R*)cdAdo, (5′*S*)cdAdoc and ^OXO^dAdo (0.5 [OD]) conversion to (5′*R*)cdIno, (5′*S*)cdInoc and ^OXO^dIno by 55 [U] of ADA during a period of 168 h at 37 °C. (**C**) The efficiency of ^OXO^dAdo (0.1 [OD]) conversion to ^OXO^dIno by different amounts of ADA in the ranges of 0.01–0.1 and 1 [U] after one minute at 37 °C. The percentages [%] on the ordinate axes represent the yield of product, either (5′*R*)/(5′*S*)cdIno or ^OXO^dIno. The dotted (●●●) lines in (**A**,**C**) represent the logarithmic trend lines. The raw (numerical) data and the values of standard deviation bars are given in Tables S1 and S2 of the Supplementary Materials. The following retention times (in minutes) of 15.09, 7.94, and 13.04 and 9.53, 3.92, and 8.83 were found for the substrates: ^OXO^dA, (5′*R*)cdA, (5′*S*)cdA and products: ^OXO^dIno, (5′*R*)cdIn, and (5′*S*)cdA, respectively. Figure 4 shows the chromatograms, which present the purity of starting materials (t_0_) and the reaction progress (percentage of product formation) over 168 h. The experiments were performed in triplicate.

This enzyme, which has been previously shown to be able to convert noncanonical 3′-deoxyadenosine to its 6-oxo derivatives, i.e., 3′-deoxyinosine [41]. Additionally, ADA recognises and converts the oxidised form of cordycepin [32]. The above adenosine deaminase activity was analysed using RP-HPLC with UV detection and, for these purposes of this study, the same strategy was applied. First, the activity of ADA was verified in the range from 1 to 11 U after 24 h at 37 °C, maintaining identical amounts of (5′*R*)cdA 6.5 μmol (0.1 [OD]). As shown in Figure 3A, the highest reaction efficiency was found for 11 U of ADA. The logarithmic trend line indicates that the enzyme concentration appears to begin to level off. Therefore, the relationship between substrate 32.9 nM (0.5 [OD]) and enzyme 55 U was studied further. It should be noted that because all the investigated molecules are derivatives of 2′-deoxyadenosine, any subtle structural differences may play a critical role. There are two main differences between cdA and ^OXO^dA: first, the additional covalent bond between C5′ and C8 renders the nucleoside structure extremely rigid (with a significant reduction in degrees of freedom) and less susceptible to external factors such as temperature [42]; second, the 8-oxo-adenine moiety of ^OXO^dAdo is able to exist in two forms, *enol* and *keto*, and can rotate around the glicosidic C1′-N9 bond, adopting a *syn* or *anti* conformation. These qualities are especially visible in the differences in retention time, given in minutes, during RP-HPLC analysis of the substrate and products.

0.5 [OD] of ^OXO^dA (5′*R*)cdA and (5′*S*)cdA were digested by 55 U of ADA for 168 h with reaction progress monitoring every 24 h. The results obtained indicate that under this condition, ^OXO^dA was converted by ADA to ^OXO^dIno within one minute (just after the addition of ADA to the dAdo solution and HPLC injection, assigned as t_0_ = 0 h), while for both diastereomers, only 5% of the substrate was digested after 24 h. The elongation time of up to 168 h resulted in yields of 34% and 32% for (5′*R*) and (5′*S*)cdIno, respectively (Figure 3B). In the control experiment, ^OXO^dAdo (0.1 [OD]) was completely deaminated after one minute in the presence of 0.7 [U] of ADA only (Figure 3C). The amount of ADA in [U] was investigated in ranges of 0.01 to 0.1 and 1.0.

**Figure 4:**
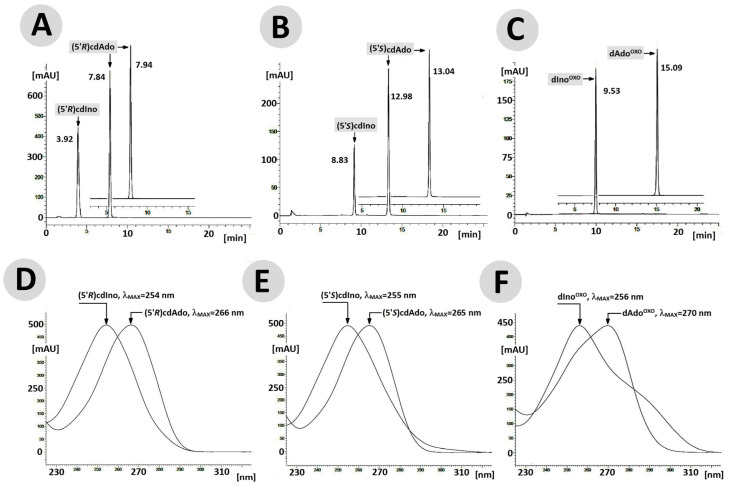
RP-HPLC chromatogram profiles recorded in the wavelength (λ) range of 190–400 nm for (5′*R*)cdAdo (**A**), (5′*S*)cdAdo (**B**), and ^OXO^dAdo (**C**) after 168 h of adenosine deaminase (55 [U]) activity. The profiles show changes in UV spectra of maximum absorption (lMAX) during the ADA-catalysed deamination of (**D**) (5′*R*)cdAdo→(5′*R*)cdIno, (**E**) (5′*S*)cdAdo→(5′*S*)cdIno, and (**F**) ^OXO^dAdo → ^OXO^dIno.

The above observation indicates that the rigid structure of the cdpurines requires a longer time for deamination by ADA (for enzyme structural accommodation), in contrast to the more flexible ^OXO^dAde (Figure 3). In all cases, the dAdo derivatives reached the active centre of the aminohydrolase, which can promote further digestion according to the mechanism presented in Figure 2. The exchange of the 6-amino group (NH_2_) of adenine by an oxygen atom derived from the H_2_O molecule and activated by the Zn^2+^ ion leads to a change in the maximum UV absorption (λ_Max)_. As presented in Figure 4D–F, in all cases, the λ_Max_ shifted in the direction of a shorter wavelength by 14 nm for ^OXO^dAdo → ^OXO^dIno, 10 nm for (5′*R*)cdAde → (5′*R*)cdIno, and 12 nm for (5′*S*)cdAde → (5′*S*)cdIno after the deamination process. The above is “diagnostic” of the discussed reaction and makes identification/monitoring easier without the need for costly HPLC MS/MS techniques. However, the use of a PDA detector that enables the measurement of absorption in abroad range of λ = 220–350 nm is required in order to determine the efficiency of the investigated reaction.

### 3.2. Deamination Product Identification of (5′R/S)cdAdo and ^OXO^dAno by MS/MS Spectroscopy Analysis

Several analytical techniques, such as an Enzyme-Linked Immunosorbent Assay (ELISA), High-Performance Liquid Chromatography (HPLC), post-digestion labelling, and Nuclear Magnetic Resonance (NMR) spectroscopy, can identify the products of these enzymatic reactions. However, each of these analytical approaches has some limitations, including an inability to identify unknown structures and the need for a large amount of the material under investigation, as in the case of 2D NMR [43,44]. With these limitations in mind, current mass spectrometry methodology is attractive, whether liquid or gas chromatography (LC or GC) is applied, especially when used in a tandem mode (MS/MS). The most commonly applied method of identifying DNA damage, and therefore nucleosides or nucleotides, is LC MS/MS, which, unlike GC, avoids the need to convert them to volatile silyl derivatives. Several groups have investigated the levels of the discussed substrates, (5′*R*/*S*)cdAdo and ^OXO^dAdo, in the genome using this method, with previous oligonucleotide enzymatic digestion [45,46,47]. Hence, in this study, high-resolution mass spectroscopy (HR MS) was used to identify and confirm the deamination products. It should be mentioned here that, although the investigated process is artificial, it is similar to what actually takes place in the cytosol and intercellular space. First, after 168 h, the compounds (substrates and reaction products) with the corresponding retention times (Figure 4) were isolated by RP-HPLC and used directly for further ESI HR MS investigation.

The raw data are available in the Supplementary Materials (SM). HR MS analysis revealed peak retention times of 15.090, 13.035, and 7.945 and 9.953, 8.826, and 3.921, in minutes, for the compounds ^OXO^dAdo, (5′*S*)cdAdo, and (5′*R*)cdAdo and ^OXO^dIno, (5′*S*)cdIno, and (5′*R*)cdIno, respectively. In the MS spectra, for ^OXO^dAdo, only the signal corresponding to 7,8-dihydro-8-oxo-adenine was observed: [M + H]^+^ *m*/*z* 268.1046 → 152.0573. On the other hand, for both diastereomers, (5′*R*)cdAdo and (5′*S*)cdAdo, characteristic fragmentation schemes of *m*/*z* 250.0942 → 164.0659 and *m*/*z* 250.0940 → 164.0659, respectively, were observed in the positive mode (ElectroSpray Ionization Mass Spectrometry analysis, Supplementary Materials) [48]. In both cases, the final fragmentation signal corresponded to the unchiral 8-hydroxymetyladenine compound.

**Figure 5:**
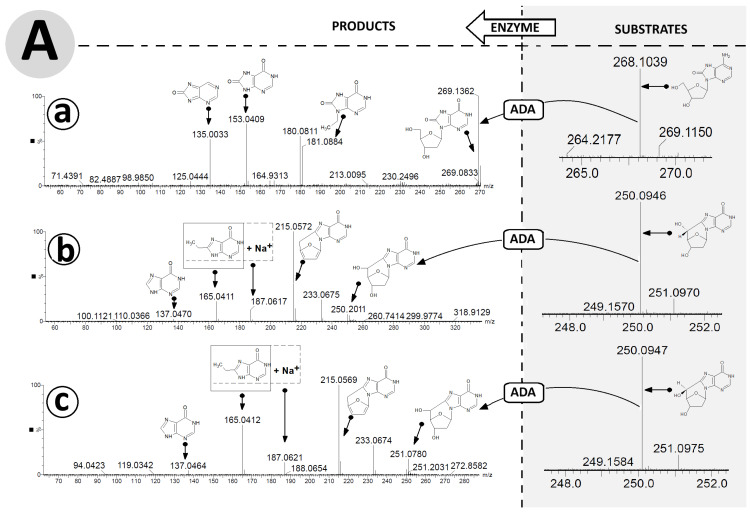

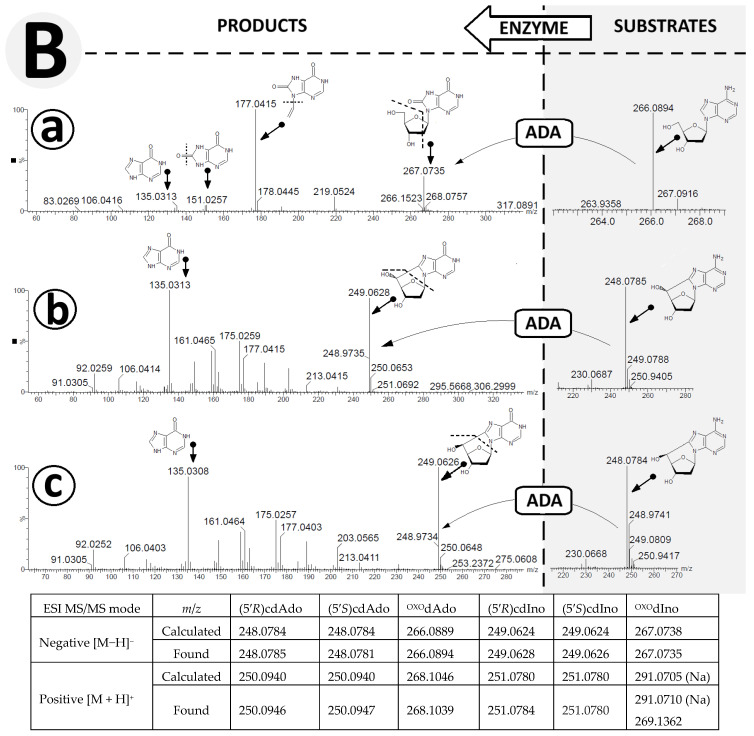
High-resolution mass spectra (HR MS) of ^OXO^dAdo, (5′*R*)cdAdo, (5′*S*)cdAdo and the corresponding reaction products ^OXO^dIno, (5′*R*)cdIno, and (5′*S*)cdIno in the (**A**) negative mode [M − H]^−^ and (**B**) positive mode [M + H]^+^. (**a**) represents changes in HR MS spectra after ^OXO^dAdo conversion to ^OXO^dIno by adenosine deaminase (ADA) action, (**b**) represents changes in HR MS spectra after (5′*R*)cdAdo conversion to (5′*R*)cdIno by ADA, (**c**) represents changes in HR MS spectra after (5′*S*)cdAdo conversion to (5′*S*)cdIno by ADA. Arrows indicate the proposed molecular structure for mass signals found in HR MS spectra. The table shows a summary of the calculated and found *m*/*z* data. Arrows: indicated the mass ion corresponding to the presented structure.

The exchange of an amino group for an oxygen atom by an aminohydrolase causes the formation of (5′*R*) 5′8-cyclo-2′-deoxyinosine, (5′*S*) 5′8-cyclo-2′-deoxyinosine, and 8-hydroxy-2′-deoxyinosine. Careful analysis of the ESI MS/MS spectra facilitated proposal of the following fragmentation path of ^OXO^dIno in positive mode: [M + H]^+^ *m*/*z* 269.1362 (^OXO^dIno) → 181.0884 (N9-etylo-8-oxo-hipoxantine) → 153.0409 (8-oxo-hipoxantine) → 135.0033 (8-oxo-purine) (Figure 5A(a)).

For both cases, analysis of the ESI MS/MS spectra of (5′*R*) and (5′*S*)cdIno revealed the same fragmentation schemes, as follows: [M + H]^+^ *m*/*z* 251.0784 ((5′*R*)cdIno) → 215.0572 (5′,3′,2′-trideoxyinosine) → 187.0617 (sodium salt of 8-ethylohipoxantine) → 165.0411 (8-ethylohipoxantine) → 137.0470 (6-oxo-purine) (Figure 5A(b,c)). It should be noted that the conversion of adenine to inosine results in different fragmentation schemes. Initially, for (5′*R*/*S*)cdInos, the dissociation of 5′ and 3′ hydroxyl groups was noted, while for (5′*R*/*S*)cdAdo, C1′-N9 glycosidic and C4′-C5′ bond cleavage was found, which forced further fragmentation paths. This observation was confirmed by the ESI MS/MS spectra analysis performed in negative ionisation mode (Figure 5B(a–c)). The following fragmentations were found: [M−H]^−^ m/z 267.0735 (^OXO^dIno) → 177.0415 (N9-ethylene-8-oxo-hipoxantine) → 151.0257 (8-OXO-hipoxantine) → 135.0313 (hipoxantine). Both diastereomers, (5′*R*) and (5′*S*) cdIno, showed the same signal patterns in the ESI MS/MS spectra collected in the negative mode [M − H], i.e., *m*/*z* 249.0628 (cdIno) → 135.03133 (hipoxantine). The results obtained confirm that both derivatives of 2′-deoxyadenosine formed by hydroxyl radical action are suitable substrates for ADA.

### 3.3. DFTB Studies of ^OXO^dAdo and (5′R/S)cdAdo Conversion to ^OXO^dIno and (5′R/S)cdIno by Adenine Deaminase

Adenosine deaminase is a specific protein that converts adenine nucleosides or their analogues to corresponding hypoxanthine derivatives in the presence of a zinc ion and H_2_O molecule [33]. The nucleophilic substitution in the purine heterocycle aromatic ring is driven by an addition–elimination reaction via the Meisenheimer intermediate, the lifetime of which limits the reaction rate (Figure 2). The *K*_m_ (Michaelis constant) values of the dAde deamination process catalysed by aminohydrolase were found at the level of 25–35 μM, with reaction rates of 190 s^−1^.

To clarify the experimental results presented above, both diastereomers of cdAdo were deemed unsuitable substrates for ADA in comparison to ^OXO^dAdo. The theoretical studies (SCC-DFTB) were performed instead of the time-consuming DFT ones in the condensed phase (Density Functional Theory) [35,49]. The DFTB does not require atom-specific description parameters, unlike molecular mechanics force fields such as UFF, AMBER, CHARM, and OPLUS [50].

**Figure 6:**
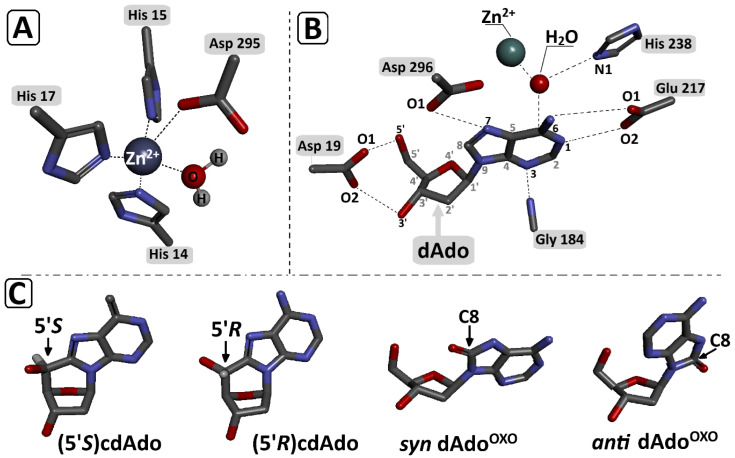
Graphical representation of (**A**) zinc ion (Zn^2+^) coordination in the enzyme (ADA) catalytic site by His 215, His 217, and Asp 295 and “activated” H_2_O; (**B**) catalytic site contacts (Michaelis complex) between dAdo and adenosine deaminase [32]; (**C**) (5′*R*)cdAdo, (5′*S*)cdAdo*, anti* ^OXO^dAdo, and *syn* ^OXO^dAdo. All spatial structures were obtained at the DFTB/3ob-3-1 level of theory in the aqueous phase and extracted from optimised Michaelis complex geometries presented on Figure 2. Red—oxygen; blue—nitrogen; grey—carbon.

Briefly, the crystal structure, i.e., 1a4L.pdb (adenosine deaminase/Pentostatin complex) was selected as a starting point [33]. Pentostatin was rearranged into OXOdAdo, (5′*R*)cdAdo, and (5′*S*)cdAdo, with subsequent Michaelis complex geometry optimisation, as previously reported [32]. It should be noted that two conformers of ^OXO^dAdo can be present in the reaction environment (*anti* and *syn*, as shown in Figure 6). Rotation of the adenine around the glycosidic bond (C1′-N9) leads to significant changes in the interaction between the adenine moiety and the ADA active site.

It has been previously found that the *syn* conformation is thermodynamically preferred to the *anti* conformation of ^OXO^dAdo, while the (5′*R*/*S*)cdAdo becomes fixed in a *syn* conformation, similar to canonical dAdo. The adenosine deaminase active centre contains a Zn^2+^ ion coordinated by His 15, His 17, His 214, Asp 296, and Asp 295 and the “activated” H_2_O molecules, which are activated by His 238. Previous crystallographic studies identified the following interactions to be crucial for the deamination reaction: Asp 19:5′ and the 3′ OH group of Ade, Gly184:N3, Glu217:N1, and N6, Asp296:N7 (Figure 5) [33].

The Zn^2+^:H_2_O and H_2_O:C6 distances of the above-mentioned bonds found in the optimised structure (SCC-DFTB) of ADA with dAdo, *syn* ^OXO^dAdo, *anti* ^OXO^dAdo, (5′*R*)cdAdo, and (5′*S*)cdAdo are presented in Table 1. For all investigated ligands except for *syn* ^OXO^dAdo, the lengths of the Zn^2+^:H_2_O and H_2_O:C6 distances were found to be fairly similar, i.e., approaching 2.12 and 2.80 Å, respectively. The rotation of adenine molecules around the glycosidic bond and adoption of the *syn* conformation (the energetically privileged form of ^OXO^dAdo) causes a significant elongation of the distance between the C6 atom of adenine and the oxygen atom of H_2_O of up to 4.03 Å. This, in turn, prevents any possibility of a nucleophilic aromatic substitution by an addition–elimination reaction (Table 1). The presence of an additional covalent bond between C5′ and C8 effectively makes the structure rigid and insusceptible to H_2_O nucleophilic attack on the C6 carbon of adenine. The interaction energy, calculated at the SCC-DFTB/3ob-3-1 level of theory, between adenosine deaminase and the above-mentioned adenosine derivatives was presented in Table 1. The above results reveal that the lowest interaction energies (IE) were found for the investigated Michaelis complexes ADA-*syn* ^OXO^dAdo and ADA-(5′*S*)cdAdo, rendering their formation less privileged than ADA-*anti* ^OXO^dAdo and ADA-(5′*R*)cdAdo. It should be noted that ADA–adenine derivative interactions warrant further systematic study at a higher level of theory (density functional theory) to shed light on the phenomenon under discussion.

## 4. Discussion

It is commonly accepted that genetic information is continuously exposed to harmful physical and chemical factors. Their effects potentially cause DNA damage, which, if unrepaired, can lead to mutagenic processes [51,52,53]. The most abundant types are ^OXO^dG, AP-sites, and single-strand breaks [54]. Due to this precarious state, cells evolved a DNA damage response system (DDR), which removes the lesioned nucleotides as modified nucleobases or as part of short oligonucleotides [55]. It should be noted that the level of free nucleotide triphosphate damage in cell is higher than that assigned in the nucleus [56]. Additionally, systematic studies have shown that around 10% of nucleosides ingested with food are incorporated into the cell genome [14,15]. Dizdaroglu et al. have shown that, depending on the preparation method, different levels of DNA damage can result [15]. Fortunately, during evolution, cells developed specific glycosylases, such as MutyH, OGG1, Nth, to limit excessive accumulation of nucleotide/nucleoside lesions in the cytosol [57]. Unfortunately, none of cdPus are not substrates for known glycosylases and can therefore be further converted to triphosphate derivatives, which can be incorporated into ds-DNA by BER polymerases. It has been shown that cdAdo can be inserted opposite Thy and dCyt, which makes it potentially mutagenic, resulting in an AT → GC transition [58].

Although 5′,8-cyclopurines are a central point of interest, their metabolism is unknown, and no data are available on their cellular secretion. Similarly, ^OXO^dAdo is considered a “forgotten” purine lesion. In contrast to the above, the ^OXO^dGuaTP has been well investigated, with some hypotheses suggesting further conversion to nucleosides by NUDT1 (nudix hydrolase 1) and nucleosidase after its release from the genome [59]. Moreover, following Dizdaroglu, it should be noted that the excretion of DNA lesions into urine as a result of diet should be excluded [15].

One of the most important enzymes involved in purine metabolism is adenosine deaminase, abundant on both sides of the cell membrane. This aminohydrolase converts adenine and its derivatives to inosine analogues [22,34]. The dysfunction or lack of ADA causes different pathological outcomes (immune deficiency syndromes) [26]. A lack of or reduced ADA activity can lead to increases in mutations as a result of the higher likelihood of lesioned dAdo being inserted into the genome. ADA acts as a guardian, and its absence leads to the accumulation of unwanted adenosine/adenine derivatives on both sides of the cell membrane. On the other hand, its high affinity for the adenine heterocycle can result in the deamination of “drugs” such as cordycepin, ultimately necessitating the administration of an ADA inhibitor (e.g., Pentostatin) [60].

Based on the above information, in this study ^OXO^dAdo and cdAdo were investigated in terms of ADA activity. Whether these adenine lesions can be metabolised to inosine explains their levels in extra- and intracellular fluids. From a medical diagnostic point of view, their presence in urine (as a convenient material for testing) can indicate the level of cancer progression, similar to ^OXO^dGuo. To the author’s knowledge, the literature contains no data regarding ADA’s interaction with DNA damage. From the perspective of purine metabolism, ADA is a crucial protein that forces the conversion of dAdo/Ado to dIno/Ino and initiates the process of uric acid formation or activates the rescue cycle of nucleoside synthesis. The latter is activated in highly proliferating cells, such as those found in cancer or in cases of malnutrition [61]. Notably, uric acid is one of the best and most common native antioxidants present in physiological conditions, with an ionisation potential lower than guanosine (IP = 6.21 versus 6.42) [62].

The results presented above indicate that adenosine deaminase isolated from calf thymus can fully convert ^OXO^dAdo into ^OXO^dIno after one minute (Figure 4C). The above results were obtained for 0.1 [OD] molecules of ^OXO^dAdo and 0.7 [U] of ADA. Previous result indicated that dAdo and Cord are deaminated effectively to dIno and 3′-deoxyinosine [32]. The exchange of the amino group for an oxygen atom reduces in retention time during RP-HPLC analysis, which indicates a decrease in the lipophilic character of the investigated molecule. The structure of ^OXO^dIno was predicted by high-resolution mass spectroscopy. The ESI MS/MS [M + H]^+^ spectra revealed that hypoxanthine is the product of ^OXO^dIno fragmentation (Figure 5A,B). Theoretical studies (SCC-DFTB calculations in the aqueous phase) indicate that the anti-conformer of ^OXO^dAdo can serve as a suitable substrate for Meisenheimer intermediate formation (Figure 2, Table 1), as in the case of dAdo [32]. In contrast, the distance between C6 and H_2_O in the ADA active centre of *syn* ^OXO^dAdo expanded up to 4.03 Å, which effectively prohibited the deamination process (Table 1). This suggests that the ratio between *syn* and *anti* conformers of ^OXO^dAdo is the limiting factor for this process.

The situation is reversed in the case of 5′8-cyclo-2′-deoxyadenosine, which exists in a fixed *syn* conformation as a result of an additional covalent bond between C5′ and C8 that inhibits flexibility. This leads to an additional chiral C5′ centre and 5′*R* and 5′*S* diastereomers. As shown previously, both diastereomers are left unrepaired by classical BER machinery—no specific glycosylases are known. Additionally, they both exert a negative impact on other DNA repair processes. Therefore, it is commonly accepted that cdAdo is repaired by the nucleotide excision repair system; however, the 5′*S* diastereomer is removed four times more slowly than the 5′*R* diastereomer [27]. These factors and the presence of cdAdo outside the genome in living organisms raise questions about its metabolism. Both chiral forms of cdAdo have been identified as substrates of adenosine deaminase. Although the structure of 5′*R*/*S* cdAdo is rigid and not susceptible to external factors such as temperature, it can be converted, albeit slowly, to the corresponding (5′*R*/*S*)cdIno. As in the case of ^OXO^dAdo, it has been observed that the N6 amino group undergoes conversion to oxygen. RP-HPLC monitoring of the deamination process shows that after 168 h, 34% and 32% of (5′*R*)cdAdo and (5′*S*)cdAdo are converted to the corresponding products (5′*R*)cdIno and (5′*S*)cdIno, respectively. This process is accompanied, as in the case of ^OXO^dAdoi → ^OXO^dIno, by a shortened retention time of (5′*R*) and (5′*S*)cdIno (the deamination product). The structures of both cdIno diastereomers were confirmed by high-resolution mass spectroscopy (ESI-HR MS, [M + H]^+^, with (5′*R*)cdIno *m*/*z* calculated at 250.0940 and found at 251.0789 and (5′*S*)cdIno *m*/*z* calculated at 250.0940 and found at 250.0946. Previous studies have already shown that analysis of tandem mass spectroscopy spectra reveals cdAdo → 8-hydroxymethylo-adenine fragmentation, i.e., [M + H]^+^ *m*/*z* 250 → 164 [48]. The crude ESI-HR MS spectra of (5′*R*) and (5′*S*) cdAdo (from this study) are presented in the Supplementary Materials. The conversion of cdAdo to cdIno by adenosine deaminase changes the fragmentation profile from glycosidic (C1′-N9) and C4′-C5′ bonds to the loss of 3′ and 5′ hydroxyl groups, followed by furan ring decomposition, giving rise to 8-ethylo-hipoxantine (Figure 5A(a,b)). Moreover, in the spectra of both diastereomers, a hypoxanthine ion was found instead of adenine. The above findings strongly indicate that ADA is able to convert cdAdo to cdIno irrespective of C5′ chirality and rigidity of the cyclopurine structure. DFTB theoretical studies show that despite the rigidity of cdAdo’s structure, the protein’s flexibility can force the deamination reaction. This process is feasible even if the distance between C6 and H_2_O is extended to 2.82 Å for (5′*R*)cdAdo and 2.92 Å for (5′*S*)cdAdo (the distance for dAdo was 2.73 Å) (Table 1). The measured differences (0.1 Å) are well supported by the RP-HPLC experimental results, which reveal that the 5′*R* diastereomer is more susceptible to N6 amino group exchange. Furthermore, the lowest energy changes between the Michaelis complex and the substrates indicate that dAdo is privileged over both cyclopurine derivatives and *anti* ^OXO^dAdo is privileged over the conformer *syn* of ^OXO^dAdo (Table 1). The results of the preliminary theoretical studies described above were obtained for the optimised ground states, where ADA and the substrate adopted mutually relaxed structures. However, to fully clarify the experimental phenomenon, further molecular dynamics studies and interaction energy calculations at a higher level are necessary.

## 5. Conclusions

Genomic DNA, the so-called seed of life, is continuously exposed to harmful external factors, both chemical and physical, which can lead to DNA damage that, if not repaired, leads to mutation and potentially carcinogenesis. Genomic DNA nucleosides and nucleotides are exposed to the same harmful factors. To protect genetic information, the damaged nucleosides and fragments of oligonucleosides removed from the genome must be converted to safe molecules and either excreted from the cell or digested. Although DNA damage repair systems have been studied, the scientific literature contains scant information about post-repair and non-genomic DNA damage utilisation mechanisms. One of the main enzymes that is abundant on both sides of the cell membrane, which regulates the homeostasis of adenine derivatives, is adenosine deaminase.

In this article, it is shown that two different kinds of damage, “isolated/simple” 7,8-dihydro-8-oxo-2′-deoxyadenosine and tandem (5′*R*/*S*) 5′,8-cyclo-2′-deoxyadenosine, are suitable substrates for adenosine deaminase (ADA). This amidohydrolase can convert ^OXO^dAdo to ^OXO^dIno very effectively. However, the presence of an additional C5′-C8 covalent bond in the dAdo structure renders cdAdo rigid and makes both diastereomers less susceptible to ADA activity. In this study, it was found that the 5′*R* diastereomer can be transformed slightly more effectively (by 3%) to cdIno than the 5′*S* diastereomer. The activity of aminohydrolase was monitored by RP-HPLC with UV detection. The structure of the reaction product was measured by high-resolution ESI MS/MS. It should be pointed out that for (5′*R*/*S*)cdIno, a different fragmentation pattern was noted than for the parent (5′*R*/*S*)cdAdo. Preliminary DFTB studies indicate that the distance between the oxygen of the activated H_2_O and C6 of (5′*R*)cdAdo is shorter by 0.1 Å than that measured for (5′*S*)cdAdo, i.e., 2.82 Å and 2.92 Å, respectively. For the *anti* ^OXO^dAdo, a measurement of 2.73 Å was recorded, which is similar to that of the native dAdo. These theoretical results provide good support for the experimental data. These findings may influence the method of measuring DNA damage levels in physiological fluids such as urine and blood using mass spectroscopy techniques. The observed levels of ^OXO^dAdo and cdAdo may be lower due to their possible conversion to ^OXO^Ino and cdIno. In light of the above, these DNA damage metabolites may be markers of oxidative stress or carcinogenesis. These findings can further shed light on the phenomenon of why the observed level of ^OXO^dAdo is much lower than that of ^OXO^dGuo in cells and physiological fluid, even though the difference in their ionisation potential is only 0.25 eV [63]. Therefore, future studies are required to investigate the metabolism of DNA damage and to identify enzymes that have remained unknown in the field of nucleic acid biochemistry.

## Figures and Tables

**Table 1:** Calculated interaction energies [Kcal mol^−1^] and distances in [Å] present in the catalytic sitec (Michaelis complexes) between adenosine deaminase and the ligands dAdo, *anti* ^OXO^dAdo, *syn* ^OXO^dAdo, (5′*R*)cdAdo, and (5′*S*)cdAdo obtained at the DFTB/3ob-3-1 level of theory in the aqueous phase (SM12) and compared with those assigned theoretically for ADA-dAdo [32].

Amino Acid of ADA	Atom Number	Ligand				
		dAdo	(5′*R*)cdAdo	(5′*S*)cdAdo	*anti* ^OXO^dAdo	*syn* ^OXO^dAdo
Asp 19 O1	O5′	2.95	2.90	2.97	2.95	3.32
Asp 19 O2	O3′	3.10	3.61	3.60	3.18	4.67
Gly 184 N	N3	3.45	3.28	3.23	3.43	6.67
Glu 217 O1	N6	3.76	4.12	4.21	3.70	6.50
Glu 217 O2	N1	3.14	3.25	3.35	3.15	8.71
His 238 N1	O (H_2_O)	2.84	2.87	2.88	2.86	2.86
Asp 296 O1	N7	3.15	3.59	3.80	3.46	5.67
Zn^2+^	O (H_2_O)	2.12	2.12	2.11	2.11	2.15
O(H_2_O)	C6	2.73	2.82	2.92	2.74	4.03
His 14 N	Zn^2+^	2.04	2.05	2.05	2.04	2.05
His 15 N		2.02	2.02	2.02	2.02	2.02
His 17 N		1.98	1.99	1.99	1.99	2.00
Asp 295 O		2.81	2.96	2.91	2.94	2.83
Enzyme	Interaction energies in Kcal mol^−1^					
ADA		29.90	30.53	30.00	47.87	39.37

## Data Availability

Data is contained within the article and Supplementary Materials.

## Supplementary Materials

The following supporting information can be downloaded at: https://www.mdpi.com/article/10.3390/cells14211665/s1, Set of ElectroSpray Ionization Mass Spectrometry (ESI MS) analysis (spectra) in positive (pos) [M + H]^+^ and negative (neg) [M − H]^−^ modes of (5′*R*)cdAdo, (5′*S*)cdAdo, 8-OXO-dAdo, (5′*R*)cdIno, (5′*S*)cdIno, 8-OXO-dIno. Set of ElectroSpray Ionization High Resolution Mass Spectrometry (ESI-HR MS) analysis (spectra) in positive (pos) [M + H]^+^ and negative (neg) [M − H]^−^ modes of (5′*R*)cdAdo, (5′*S*)cdAdo, 8-OXO-dAdo, (5′*R*)cdIno, (5′*S*)cdIno, 8-OXO-dIno. Set of ElectroSpray Ionization Tandem Mass Spectrometry (ESI MS/MS) analysis (spectra) in positive (pos) [M + H]^+^ and negative (neg) [M − H]^−^ modes of (5′*R*)cdAdo, (5′*S*)cdAdo, 8-OXO-dAdo, (5′*R*)cdIno, (5′*S*)cdIno, 8-OXO-dIno; Table S1. Raw data of nucleoside ((5′*R*)cdAdo, (5′*S*)cdAdo, ^OXO^dAdo) digestion by adenosine deaminase, monitored by RP-HPLC (λ = 260 nm) analysis; Table S2. Average value, given in percentage [%] and the standard deviation of nucleoside digestion by adenosine deaminase; Table S3. The energies (Hatrre) of Adenosine demainase (ADA), investigated nucleosides and formed Michaelis complex, calculated at DFTB/3ob-3-1 level of theory in the aqueous phase (SM12); Optimized *.pdb structures of adenosine deaminase (ADA) with: (5′*R*)cdAdo ((5*R*)cdAdo_ADA.pdb), (5′*S*)cdAdo ((5*S*)cdAdo_ADA.pdb), *syn* ^OXO^dAdo (syn_oxodAdo_ADA.pdb) and a*nti* ^OXO^dAdo (anti_oxodAdo_ADA.pdb).
