# Supplementary material for: 5′,8-cyclo-dAdo and 8-oxo-dAdo DNA Lesions Are Both Substrates of Adenosine Deaminase: A Preliminary Study
Source: Cells. 2025 Oct 23;14(21):1665. doi: 10.3390/cells14211665 (PMC12607335; doi:10.3390/cells14211665)

Single Mass Analysis
Tolerance = 5.0 PPM / DBE: min = -1.5, max = 150.0
Element prediction: Off
Number of isotope peaks used for i-FIT = 9

Monoisotopic Mass, Even Electron Ions
207 formula(e) evaluated with 2 results within limits (all results (up to 1000) for each mass)
Elements Used:
C: 0-60 H: 0-50 N: 1-5 O: 0-6 Na: 0-1

250709_BK_R2_pos_ACN_ 14 (0.160) Cm (14:16-(6:8+35:39)) 1: TOF MS ES+ 8.18e+006

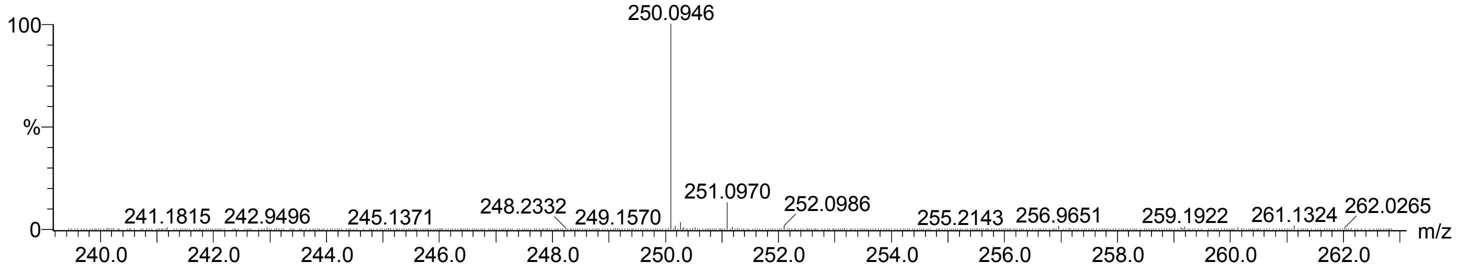

Minimum:				-1.5				
Maximum:		5.0	5.0	150.0				
Mass	Calc. Mass	mDa	PPM	DBE	i-FIT	Norm	Conf(%)	Formula
250.0946	250.0940	0.6	2.4	7.5	2579.5	0.002	99.83	C10 H12 N5 O3
	250.0956	-1.0	-4.0	8.5	2585.8	6.373	0.17	C13 H13 N3 O Na

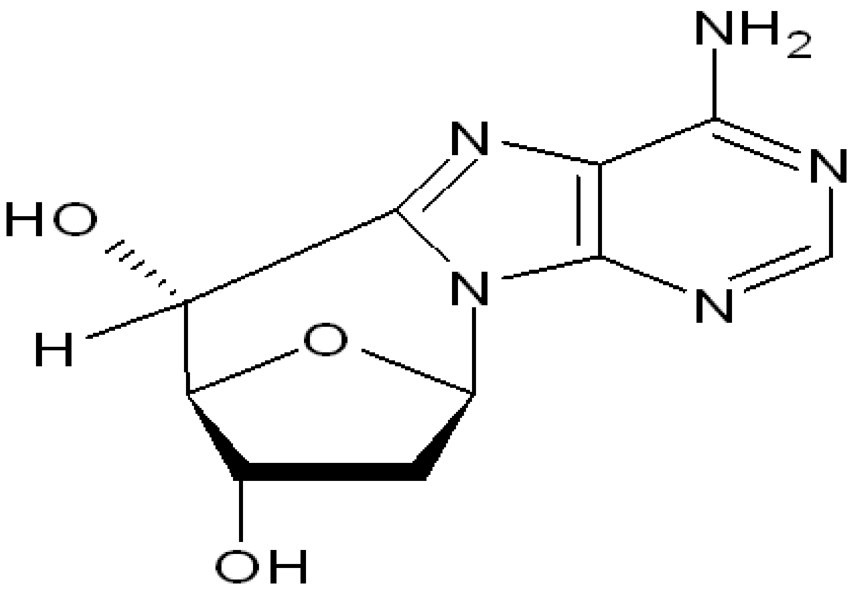

Supplement: Supplementary file 1 [file cells-14-01665-s001.zip › HR MS spectra/(5R)cdAdo_esi_HRMS_pos_250.pdf]
