# Supplementary material for: 5′,8-cyclo-dAdo and 8-oxo-dAdo DNA Lesions Are Both Substrates of Adenosine Deaminase: A Preliminary Study
Source: Cells. 2025 Oct 23;14(21):1665. doi: 10.3390/cells14211665 (PMC12607335; doi:10.3390/cells14211665)

Elemental Composition Report

Single Mass Analysis

Tolerance = 5.0 PPM / DBE: min = -1.5, max = 150.0
Element prediction: Off
Number of isotope peaks used for i-FIT = 9

Monoisotopic Mass, Even Electron Ions
207 formula(e) evaluated with 2 results within limits (all results (up to 1000) for each mass)
Elements Used:
C: 0-60 H: 0-50 N: 1-5 O: 0-6 Na: 0-1

250709_BK_R1_pos_ACN_A 16 (0.177) Cm (16:23-9) TOF MS ES+ 7.03e+005

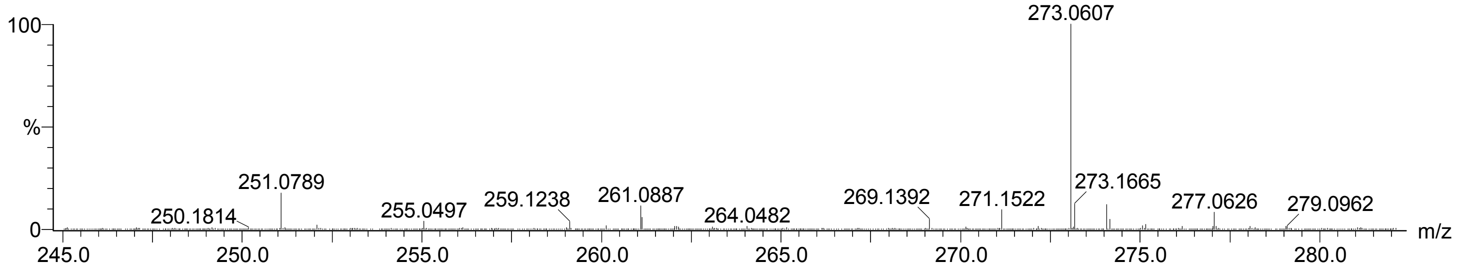

Minimum:				-1.5				
Maximum:		5.0	5.0	150.0				
Mass	Calc. Mass	mDa	PPM	DBE	i-FIT	Norm	Conf(%)	Formula
251.0789	251.0780	0.9	3.6	7.5	1929.5	0.460	63.12	C10 H11 N4 O4
	251.0796	-0.7	-2.8	8.5	1930.0	0.998	36.88	C13 H12 N2 O2 Na

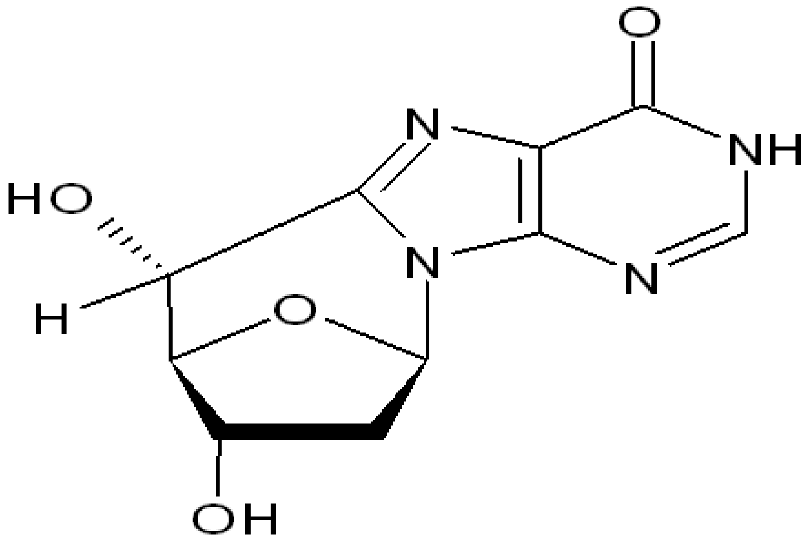

Supplement: Supplementary file 1 [file cells-14-01665-s001.zip › HR MS spectra/(5R)cdIno_esi_HRMS_pos_251.pdf]
