# Supplementary material for: 5′,8-cyclo-dAdo and 8-oxo-dAdo DNA Lesions Are Both Substrates of Adenosine Deaminase: A Preliminary Study
Source: Cells. 2025 Oct 23;14(21):1665. doi: 10.3390/cells14211665 (PMC12607335; doi:10.3390/cells14211665)

Elemental Composition Report

Single Mass Analysis

Tolerance = 5.0 PPM / DBE: min = -1.5, max = 150.0
Element prediction: Off
Number of isotope peaks used for i-FIT = 9

Monoisotopic Mass, Even Electron Ions
207 formula(e) evaluated with 2 results within limits (all results (up to 1000) for each mass)
Elements Used:
C: 0-60 H: 0-50 N: 1-5 O: 0-6 Na: 0-1

250709_BK_S3_pos_ACN_ 18 (0.205) Cm (15:18-(41:49+4:8)) 1: TOF MS ES+ 8.85e+006

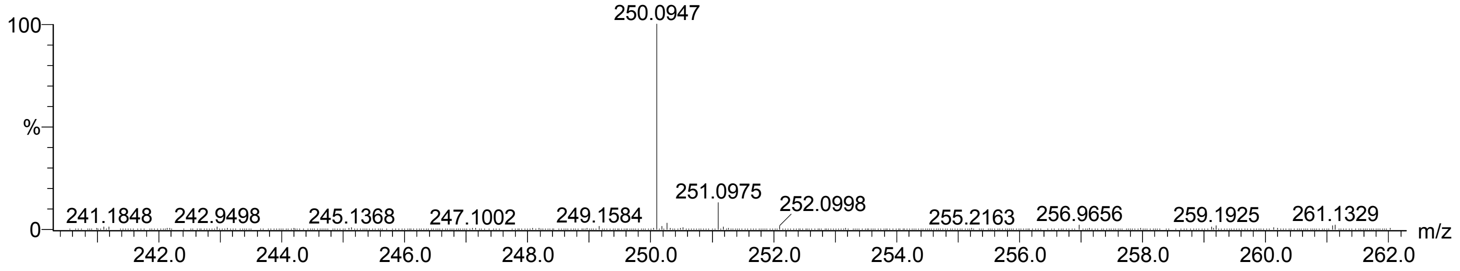

Minimum:				-1.5				
Maximum:		5.0	5.0	150.0				
Mass	Calc. Mass	mDa	PPM	DBE	i-FIT	Norm	Conf(%)	Formula
250.0947	250.0940	0.7	2.8	7.5	2645.5	0.009	99.13	C10 H12 N5 O3
	250.0956	-0.9	-3.6	8.5	2650.2	4.741	0.87	C13 H13 N3 O Na

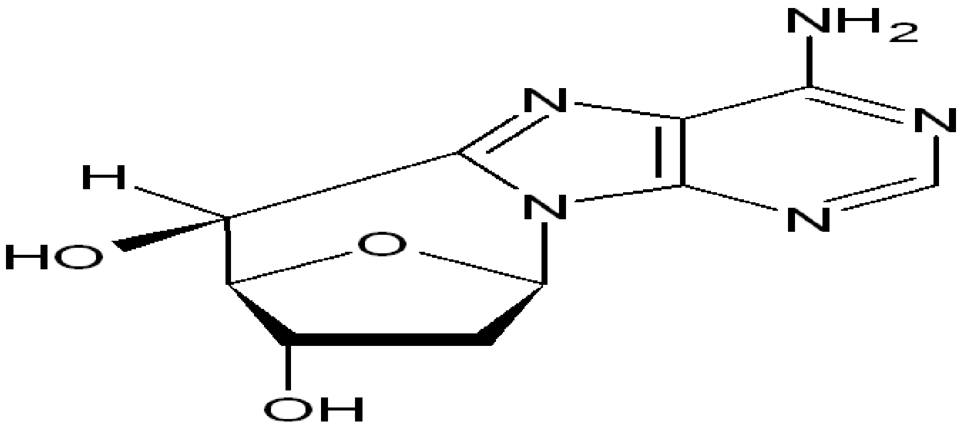

Supplement: Supplementary file 1 [file cells-14-01665-s001.zip › HR MS spectra/(5S)cdAdo_esi_HRMS_pos_250.pdf]
