# Supplementary material for: 5′,8-cyclo-dAdo and 8-oxo-dAdo DNA Lesions Are Both Substrates of Adenosine Deaminase: A Preliminary Study
Source: Cells. 2025 Oct 23;14(21):1665. doi: 10.3390/cells14211665 (PMC12607335; doi:10.3390/cells14211665)

Elemental Composition Report

Single Mass Analysis

Tolerance = 5.0 PPM / DBE: min = -1.5, max = 150.0
Element prediction: Off
Number of isotope peaks used for i-FIT = 9

Monoisotopic Mass, Even Electron Ions
270 formula(e) evaluated with 1 results within limits (all results (up to 1000) for each mass)
Elements Used:
C: 0-60 H: 0-50 N: 1-5 O: 0-9 Na: 0-1

250709_BK_S2_neg_ACN_A 35 (0.374) Cm (35:41-6:10) TOF MS ES- 4.84e+005

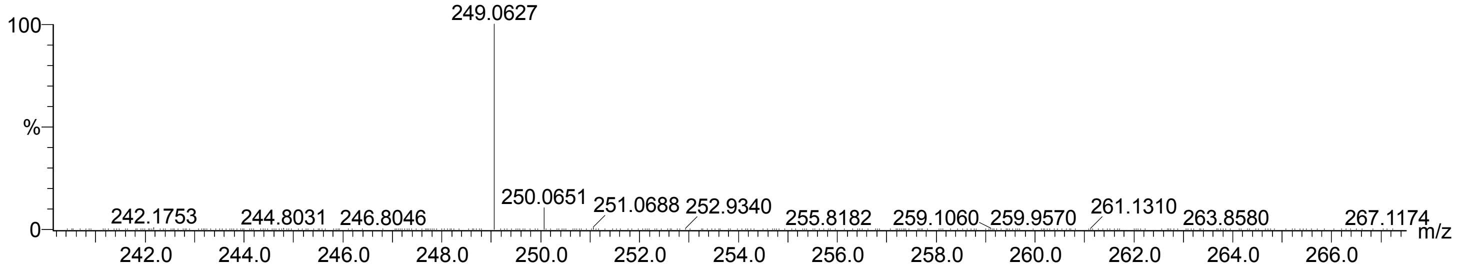

Mass	Calc. Mass	mDa	PPM	DBE	i-FIT	Norm	Conf(%)	Formula
Minimum:				-1.5				
Maximum:		5.0	5.0	150.0				
249.0627	249.0624	0.3	1.2	8.5	810.4	n/a	n/a	C10 H9 N4 O4

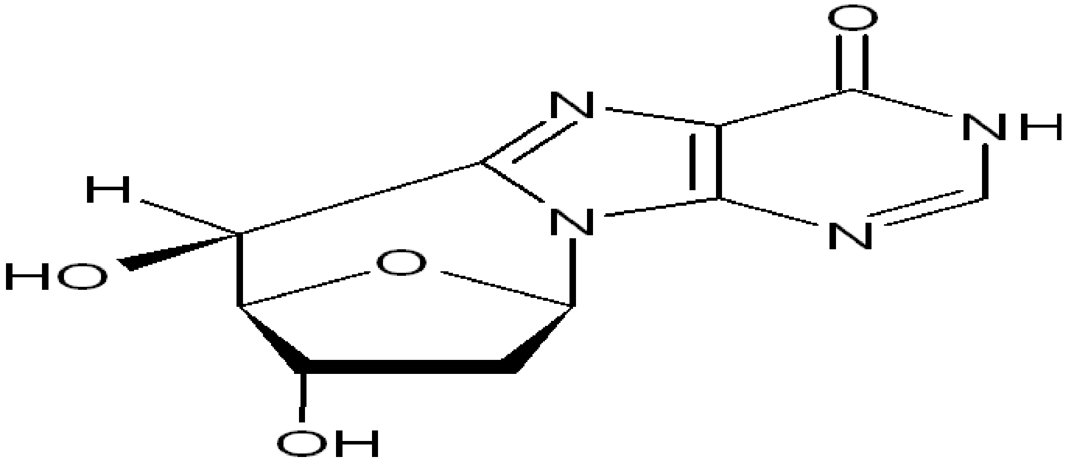

Supplement: Supplementary file 1 [file cells-14-01665-s001.zip › HR MS spectra/(5S)cdIno_esi_HRMS_neg_249.pdf]
