# Supplementary material for: 5′,8-cyclo-dAdo and 8-oxo-dAdo DNA Lesions Are Both Substrates of Adenosine Deaminase: A Preliminary Study
Source: Cells. 2025 Oct 23;14(21):1665. doi: 10.3390/cells14211665 (PMC12607335; doi:10.3390/cells14211665)

Elemental Composition Report

Single Mass Analysis

Tolerance = 5.0 PPM / DBE: min = -1.5, max = 150.0
Element prediction: Off
Number of isotope peaks used for i-FIT = 9

Monoisotopic Mass, Even Electron Ions
285 formula(e) evaluated with 2 results within limits (all results (up to 1000) for each mass)
Elements Used:
C: 0-60 H: 0-50 N: 1-5 O: 0-9 Na: 0-1
250709_BK_O2_neg_ACN_A 37 (0.391) Cm (37:46-3:8)

TOF MS ES-
2.82e+006

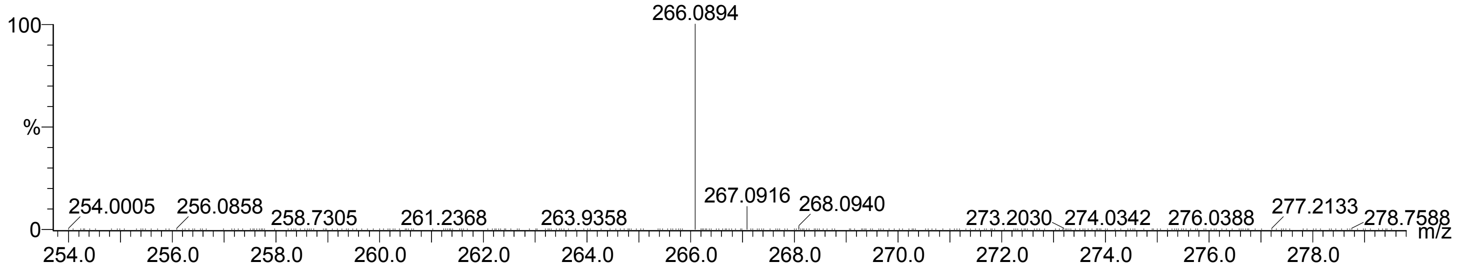

Minimum:				-1.5				
Maximum:		5.0	5.0	150.0				
Mass	Calc. Mass	mDa	PPM	DBE	i-FIT	Norm	Conf(%)	Formula
266.0894	266.0889	0.5	1.9	7.5	925.4	0.032	96.86	C10 H12 N5 O4
	266.0905	-1.1	-4.1	8.5	928.8	3.462	3.14	C13 H13 N3 O2 Na

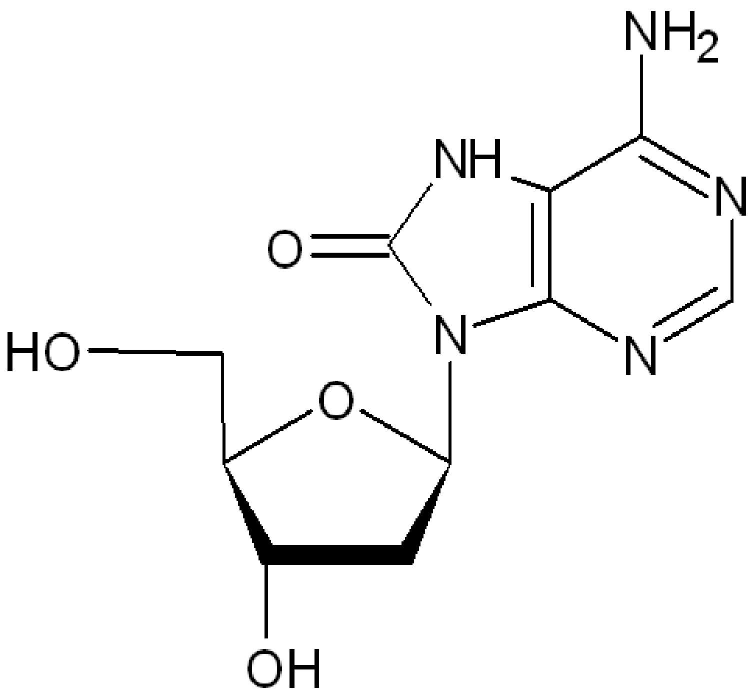

Supplement: Supplementary file 1 [file cells-14-01665-s001.zip › HR MS spectra/8oxodAdo_esi_HRMS_neg_266.pdf]
