# Supplementary material for: 5′,8-cyclo-dAdo and 8-oxo-dAdo DNA Lesions Are Both Substrates of Adenosine Deaminase: A Preliminary Study
Source: Cells. 2025 Oct 23;14(21):1665. doi: 10.3390/cells14211665 (PMC12607335; doi:10.3390/cells14211665)

Elemental Composition Report

Single Mass Analysis

Tolerance = 5.0 PPM / DBE: min = -1.5, max = 150.0
Element prediction: Off
Number of isotope peaks used for i-FIT = 9

Monoisotopic Mass, Even Electron Ions
157 formula(e) evaluated with 1 results within limits (all results (up to 1000) for each mass)
Elements Used:
C: 0-60 H: 0-50 N: 1-5 O: 0-9

250709_BK_O1_neg_ACN_A 14 (0.159) Cm (11:16-(46:53+3:7)) TOF MS ES- 2.33e+006

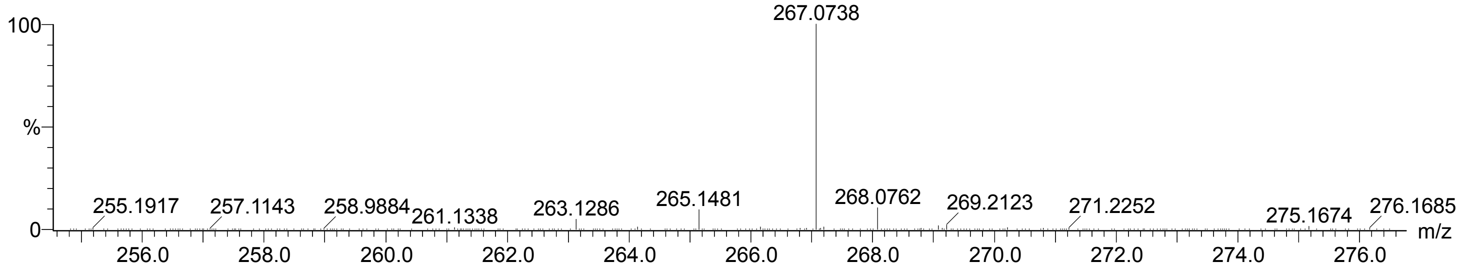

	Mass	Calc. Mass	mDa	PPM	DBE	i-FIT	Norm	Conf(%)	Formula
Minimum:					-1.5				
Maximum:			5.0	5.0	150.0				
	267.0738	267.0729	0.9	3.4	7.5	1200.0	n/a	n/a	C10 H11 N4 O5

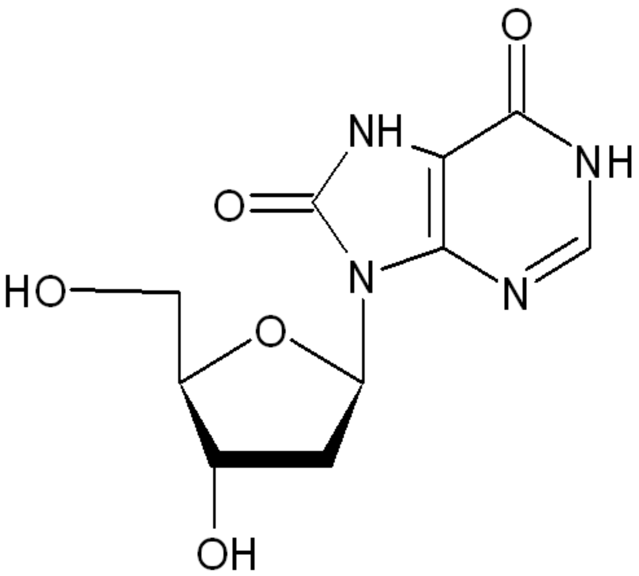

Supplement: Supplementary file 1 [file cells-14-01665-s001.zip › HR MS spectra/8oxodAdo_esi_HRMS_neg_267.pdf]
