# Supplementary material for: 5′,8-cyclo-dAdo and 8-oxo-dAdo DNA Lesions Are Both Substrates of Adenosine Deaminase: A Preliminary Study
Source: Cells. 2025 Oct 23;14(21):1665. doi: 10.3390/cells14211665 (PMC12607335; doi:10.3390/cells14211665)
Supplement: Supplementary file 1 [file cells-14-01665-s001.zip › Table S1, S2 and S3.pdf]

Table S1. Raw data of dIno derivatives formation by adenosine deaminase (ADA) monitored by RP-HPLC (λ=260 nm) analysis.

The calculated percentage [%] of dIno derivatives formation by 55 [U] of adenosine deaminase (ADA) monitored by RP-HPLC analysis at λ=260 nm.									
Time in hours	Repetition 1			Repetition 2			Repetition 3		
	(5’*S*)cdIno	(5’*R*)cdIno	^{oxo}dIno	(5’*S*)cdIno	(5’*R*)cdIno	^{oxo}dIno	(5’*S*)cdIno	(5’*R*)cdIno	^{oxo}dIno
0	0	0	0	0	0	0	0	0	0
24	5.1	5.3	100.00	5.11	5.47	100.00	5.91	5.95	100.00
48	9.89	10.53	100.00	9.76	11.21	100.00	11.09	11.61	100.00
72	14.5	15.42	100.00	14.75	16.32	100.00	16.3	16.71	100.00
96	18.7	18.99	100.00	19.65	21.67	100.00	19.57	20.86	100.00
120	23.11c	24.92	100.00	23.53	25.59	100.00	23.75	24.18	100.00
144	26.98	29.31	100.00	26.9	29.91	100.00	27.55	29.84	100.00
168	33.16	33.67	100.00	31.87	34.54	100.00	30.57	34.13	100.00

Raw data of dIno derivatives formation by different adenosine deaminase (ADA) amounts, monitored by RP-HPLC (λ=260 nm) analysis.				
ADA [U]	(5’*R*)cdAdo → (5’*R*)cdIno after 24 hours		ADA [U]	^{oxo}dAdo→^{oxo}dIno after 1 minute [%]
	(5’*R*)cdIno [%]			^{oxo}dIno
0	0.00		0	0.00
1	0.56		0.01	29.30
2	0.91		0.02	58.20
3	1.21		0.03	67.80
4	1.28		0.04	76.50
5	1.55		0.05	88.20
6	1.80		0.06	95.40
7	1.90		0.07	100.00
8	1.99		0.08	100.00
9	2.27		0.09	100.00
10	2.36		0.1	100.00
11	0.00		1	100.00

Table S2. Average value, given in percentage [%] and the standard deviation of nucleoside digestion by adenosine deaminase (ADA).

Hours	The average value, given in percentage [%] of dIno derivatives formation by adenosine deaminase 55 [U]			The standard deviation		
	(5'*S*)cdIno	(5'*R*)cdIno	^{OXO}dIno	(5'*S*)cdIno	(5'*R*)cdIno	^{OXO}dIno
0.00	0	0	0	0	0	0
24.00	5.37	5.57	100.00	0.46	0.34	0.00
48.00	10.25	11.12	100.00	0.73	0.55	0.00
72.00	15.18	16.15	100.00	0.98	0.66	0.00
96.00	19.31	20.51	100.00	0.53	1.37	0.00
120.00	23.46	24.90	100.00	0.33	0.71	0.00
144.00	27.14	29.69	100.00	0.35	0.33	0.00
168.00	31.87	34.11	100.00	1.30	0.44	0.00

Table S3. The energies (Hatrre) of Adenosine demainase (ADA), investigated nucleosides and formed Michaelis complex, calculated at DFTB/3ob-3-1 level of theory in the aqueous phase (SM12)

Nucleoside		MIchaelis complex	Adenosine deaminase
syn^{OXO}**dAdo**	-47,34405487	-7344,787391	-7297,380594
anti^{OXO}**dAdo**	-47,34123226	-7344,837660	-7297,420145
**(5'*R*)cdAdo**	-43,21207538	-7340,746938	-7297,486216
**(5'*S*)cdAdo**	-43,21409827	-7340,817466	-7297,555560
**dAdo**	-43,96241040	-7341,362708	-7297,352657
