# Supplementary figures and images for: 5′,8-cyclo-dAdo and 8-oxo-dAdo DNA Lesions Are Both Substrates of Adenosine Deaminase: A Preliminary Study
Source: Cells. 2025 Oct 23;14(21):1665. doi: 10.3390/cells14211665 (PMC12607335; doi:10.3390/cells14211665)

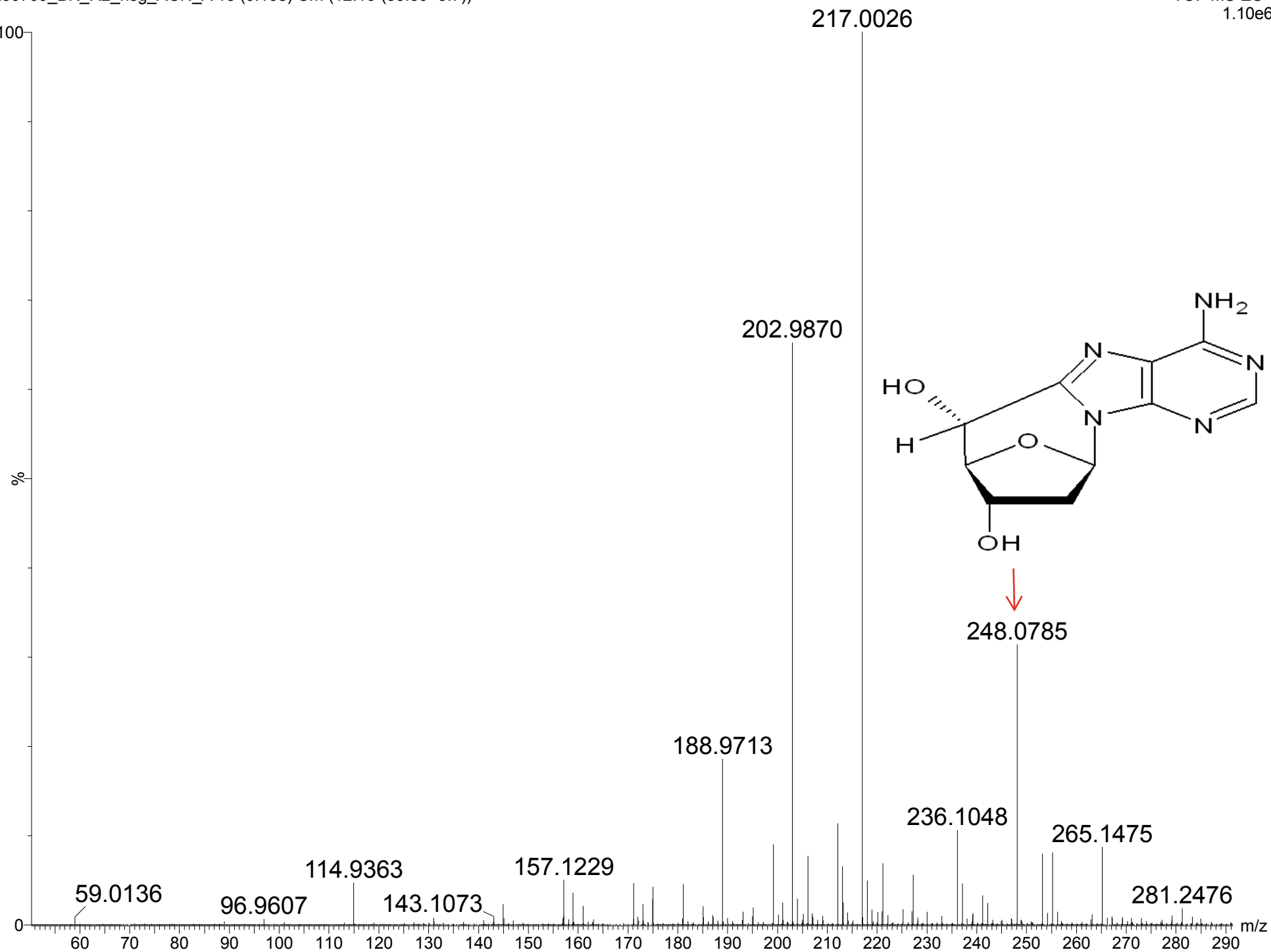

Supplement: Supplementary file 1 [file cells-14-01665-s001.zip › ESI MS spectra/(5R)cdAdo_esi_spec_neg.pdf]

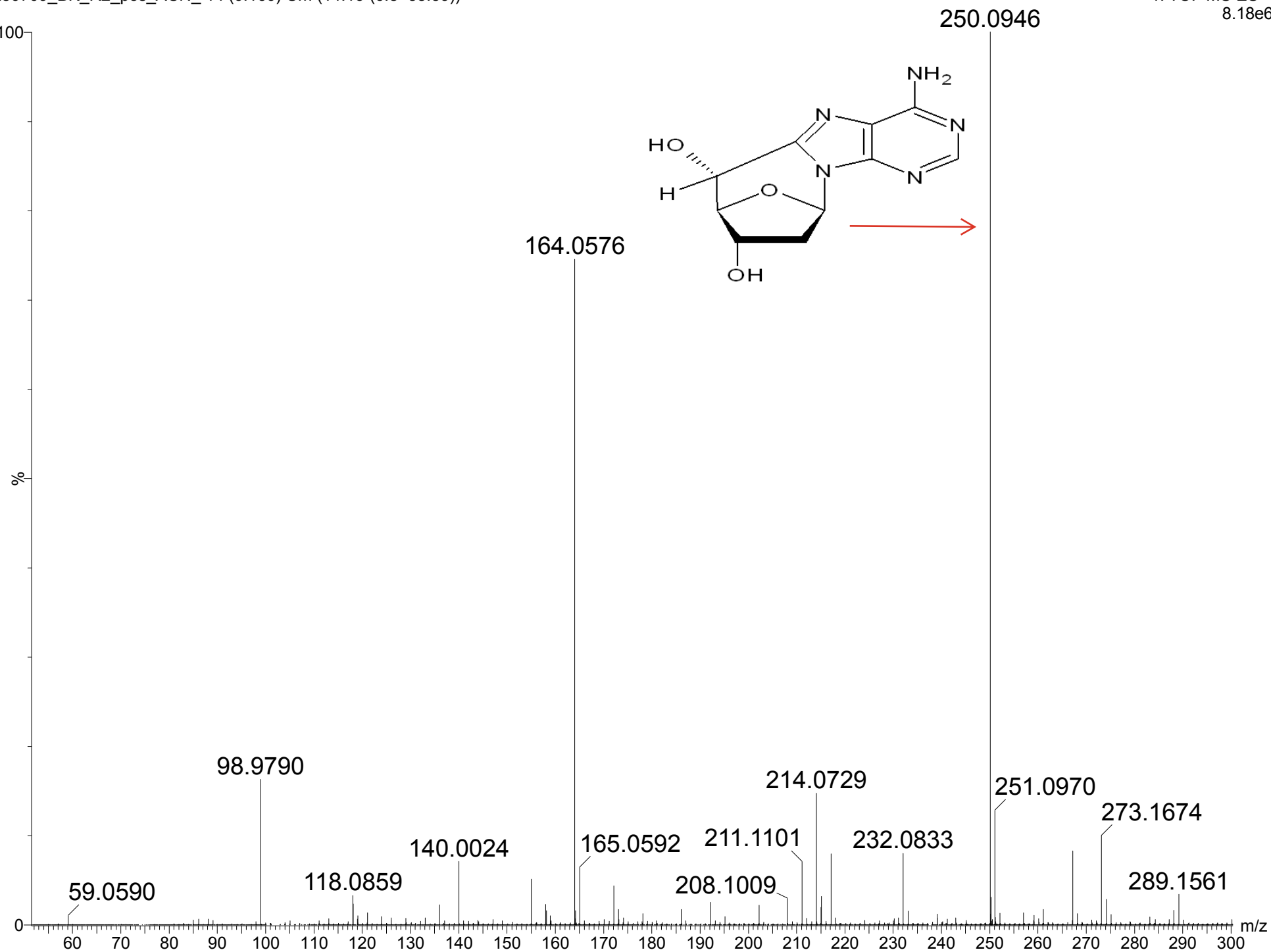

Supplement: Supplementary file 1 [file cells-14-01665-s001.zip › ESI MS spectra/(5R)cdAdo_esi_spec_pos.pdf]

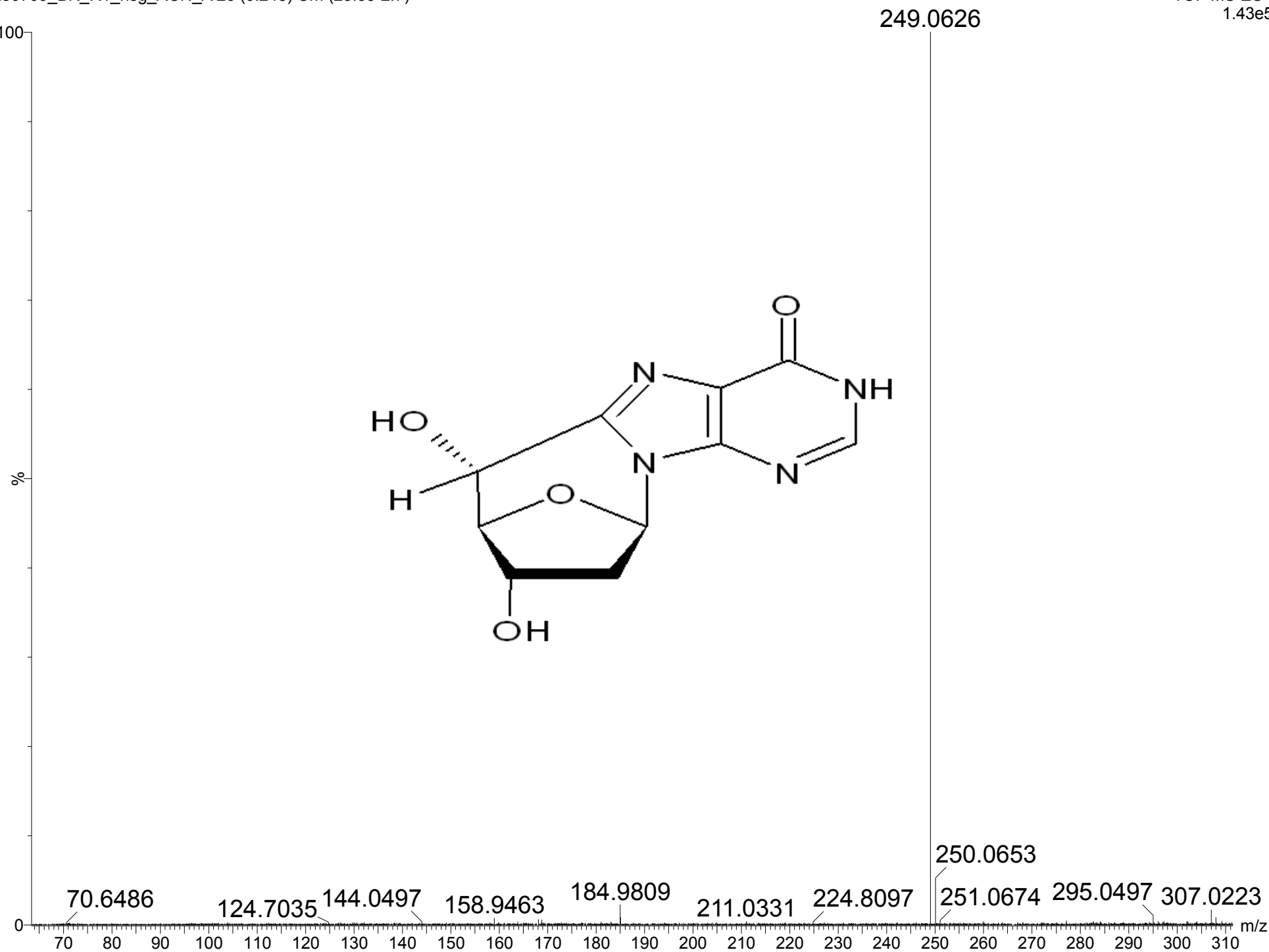

Supplement: Supplementary file 1 [file cells-14-01665-s001.zip › ESI MS spectra/(5R)cdIno_esi_spec_neg.pdf]

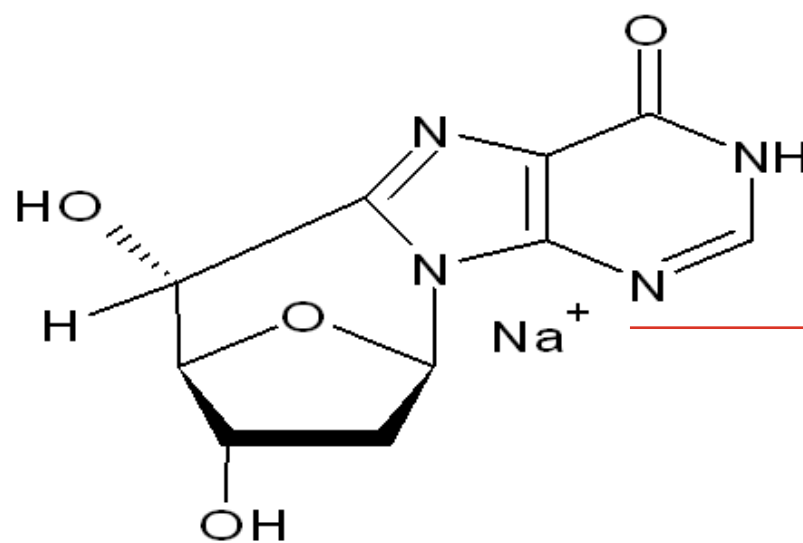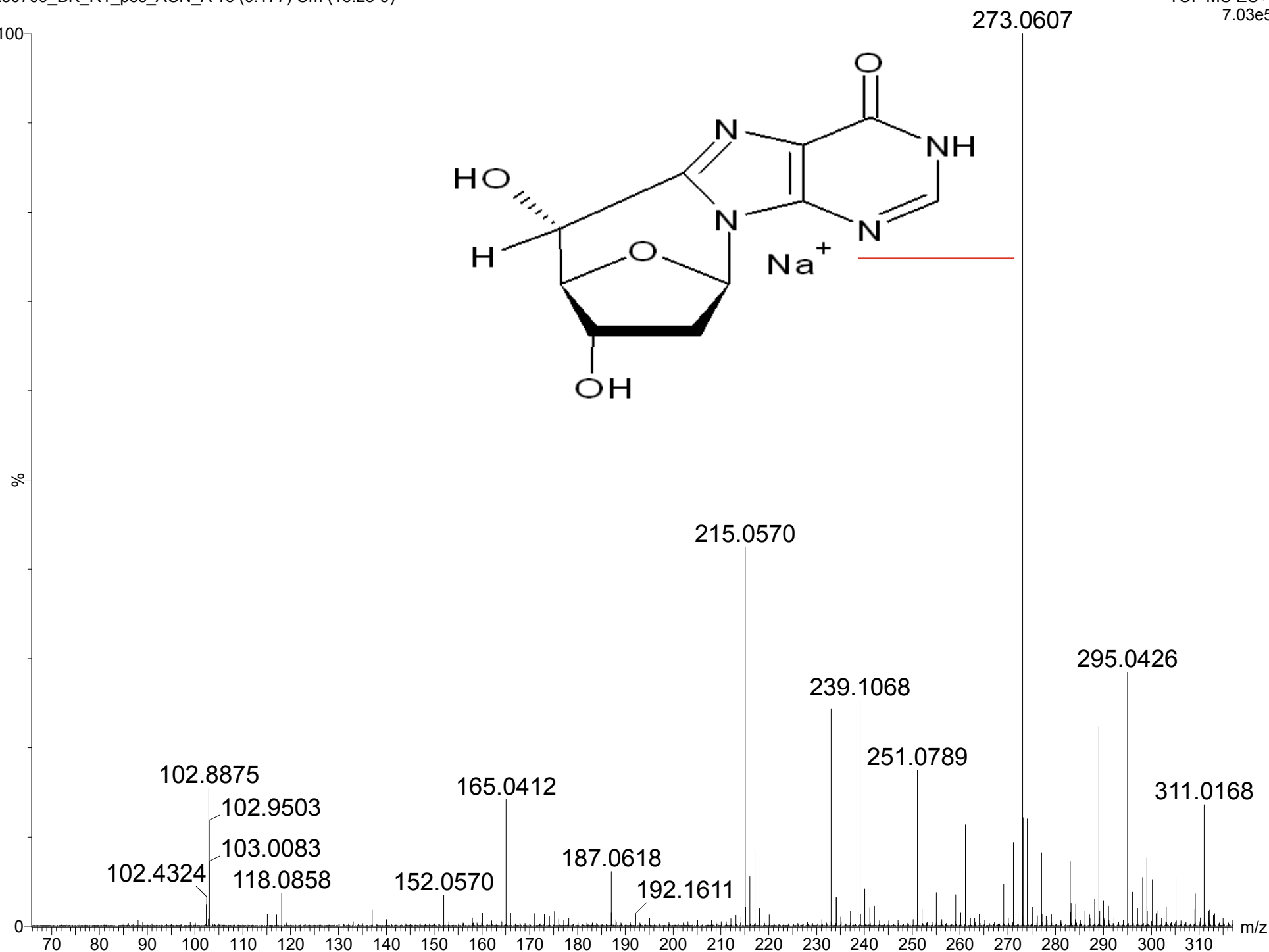

Supplement: Supplementary file 1 [file cells-14-01665-s001.zip › ESI MS spectra/(5R)cdIno_esi_spec_pos.pdf]

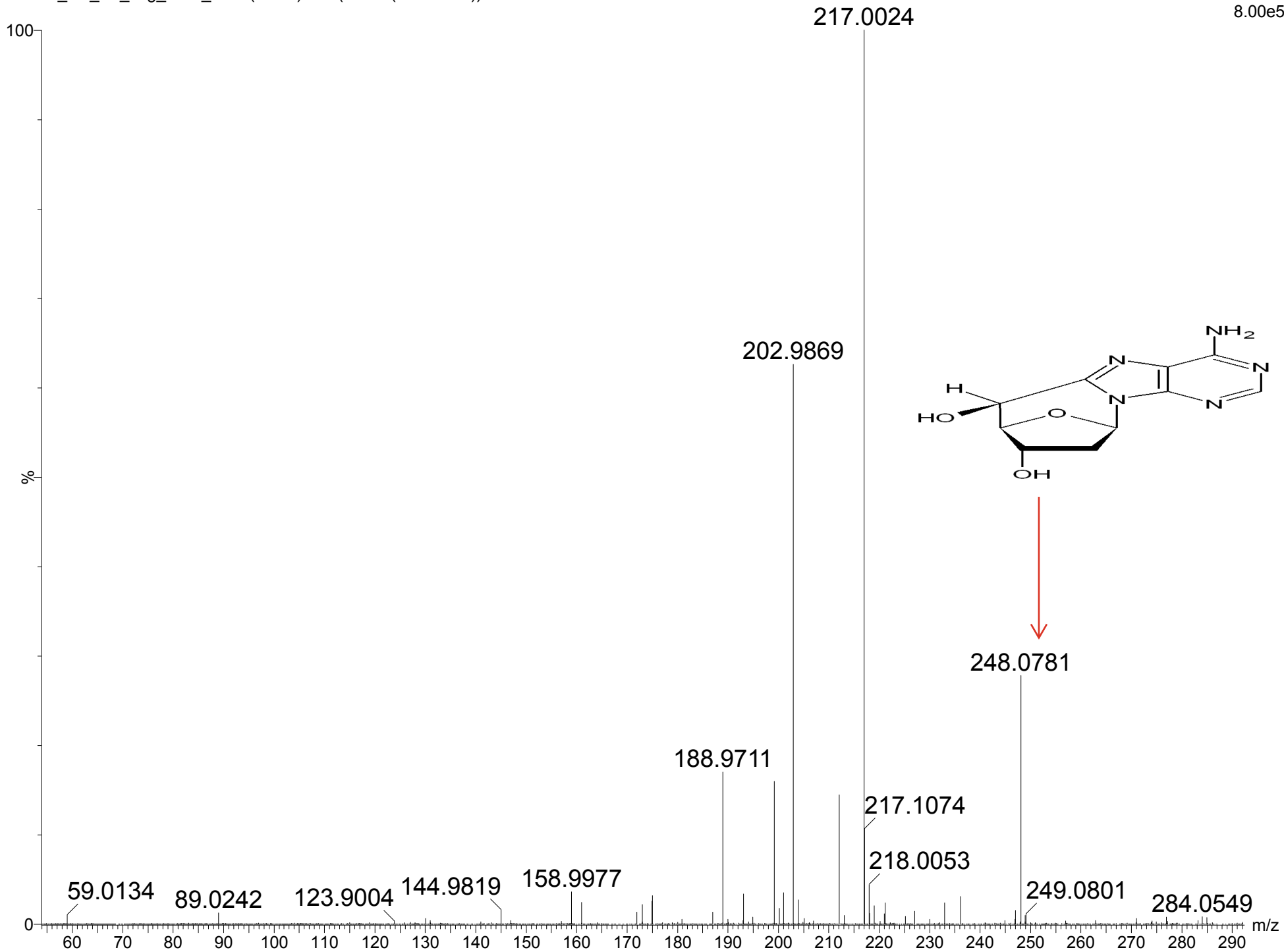

Supplement: Supplementary file 1 [file cells-14-01665-s001.zip › ESI MS spectra/(5S)cdAdo_esi_spec_neg.pdf]

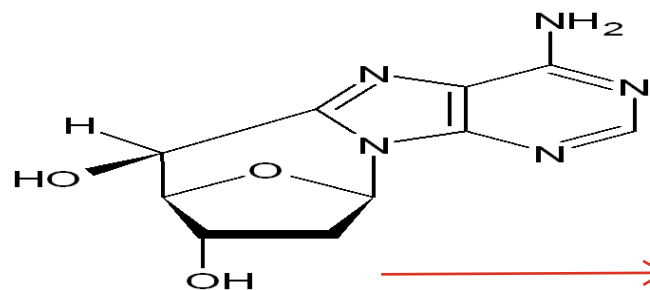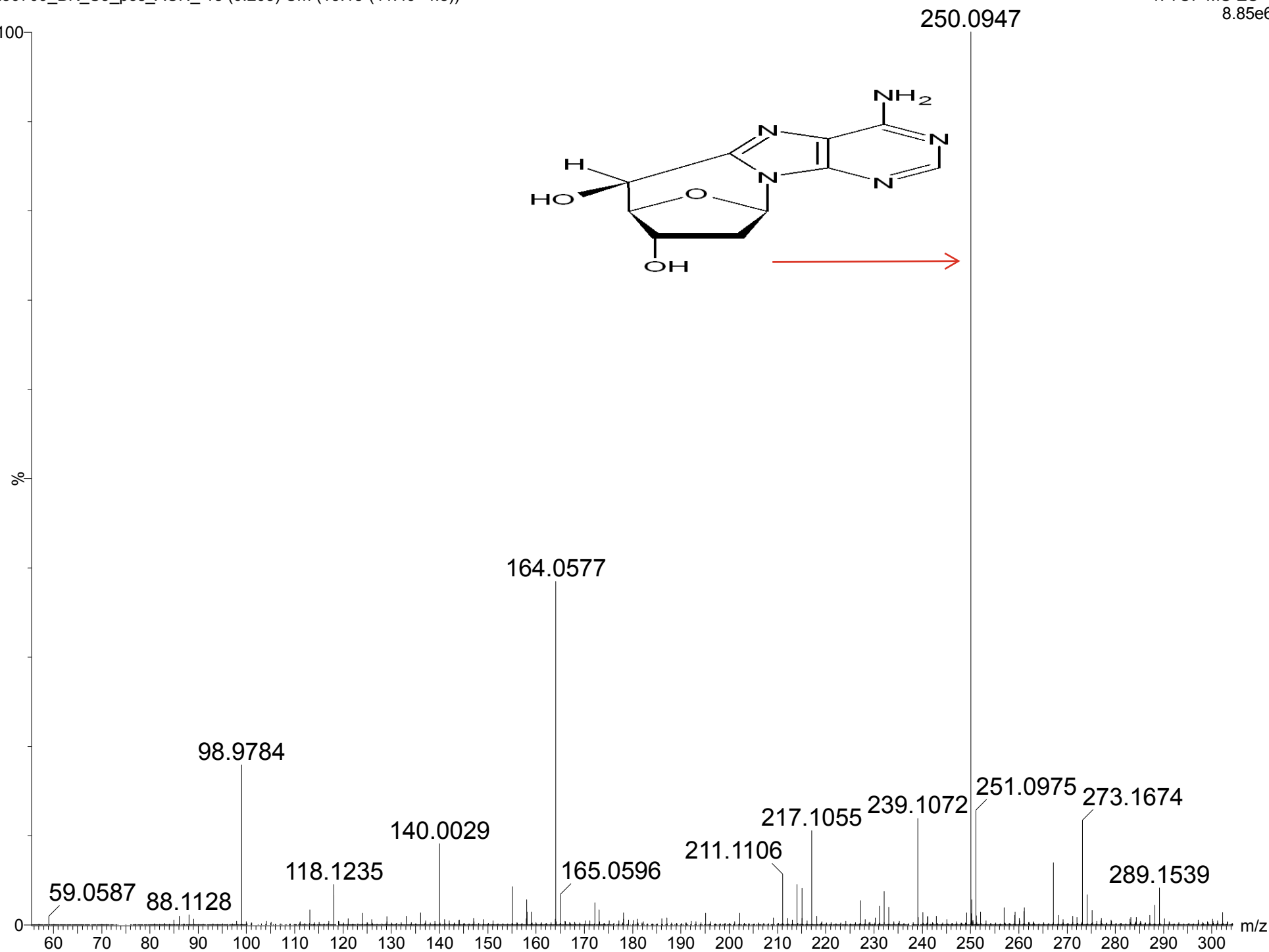

Supplement: Supplementary file 1 [file cells-14-01665-s001.zip › ESI MS spectra/(5S)cdAdo_esi_spec_pos.pdf]

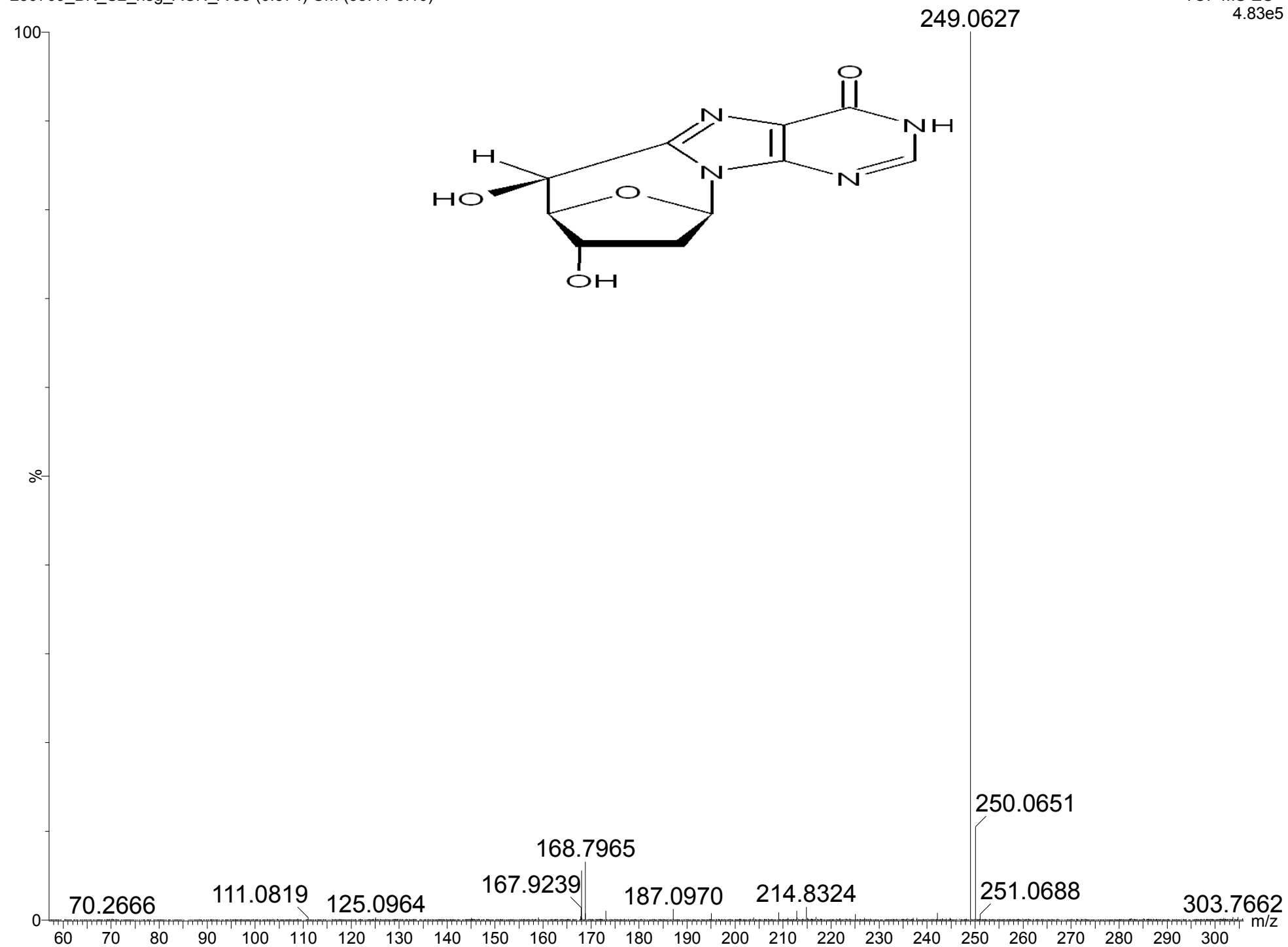

Supplement: Supplementary file 1 [file cells-14-01665-s001.zip › ESI MS spectra/(5S)cdIno_esi_spec_neg.pdf]

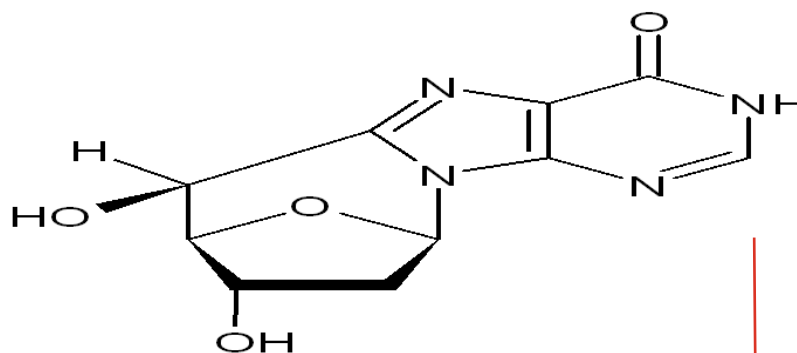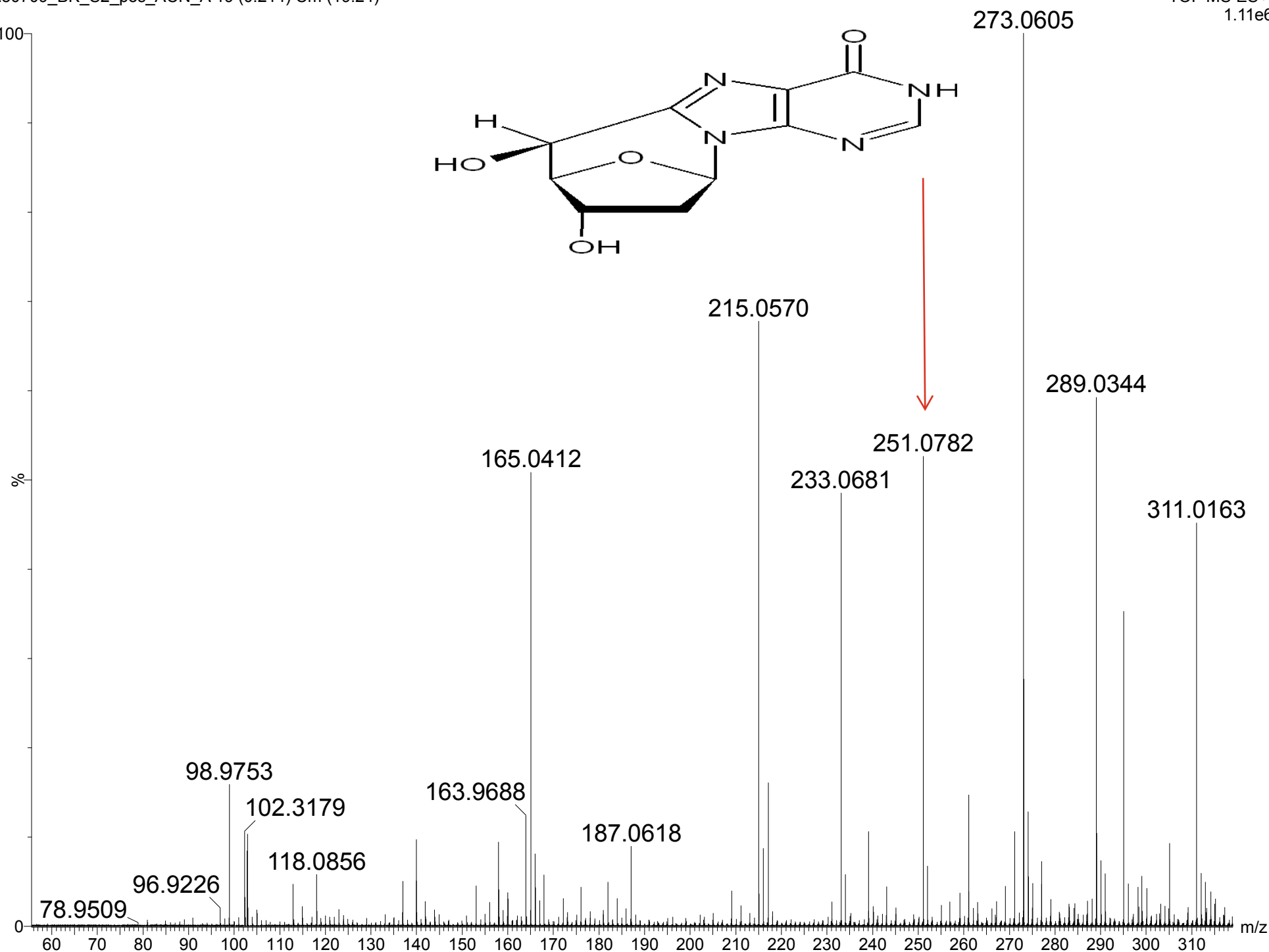

Supplement: Supplementary file 1 [file cells-14-01665-s001.zip › ESI MS spectra/(5S)cdIno_esi_spec_pos.pdf]

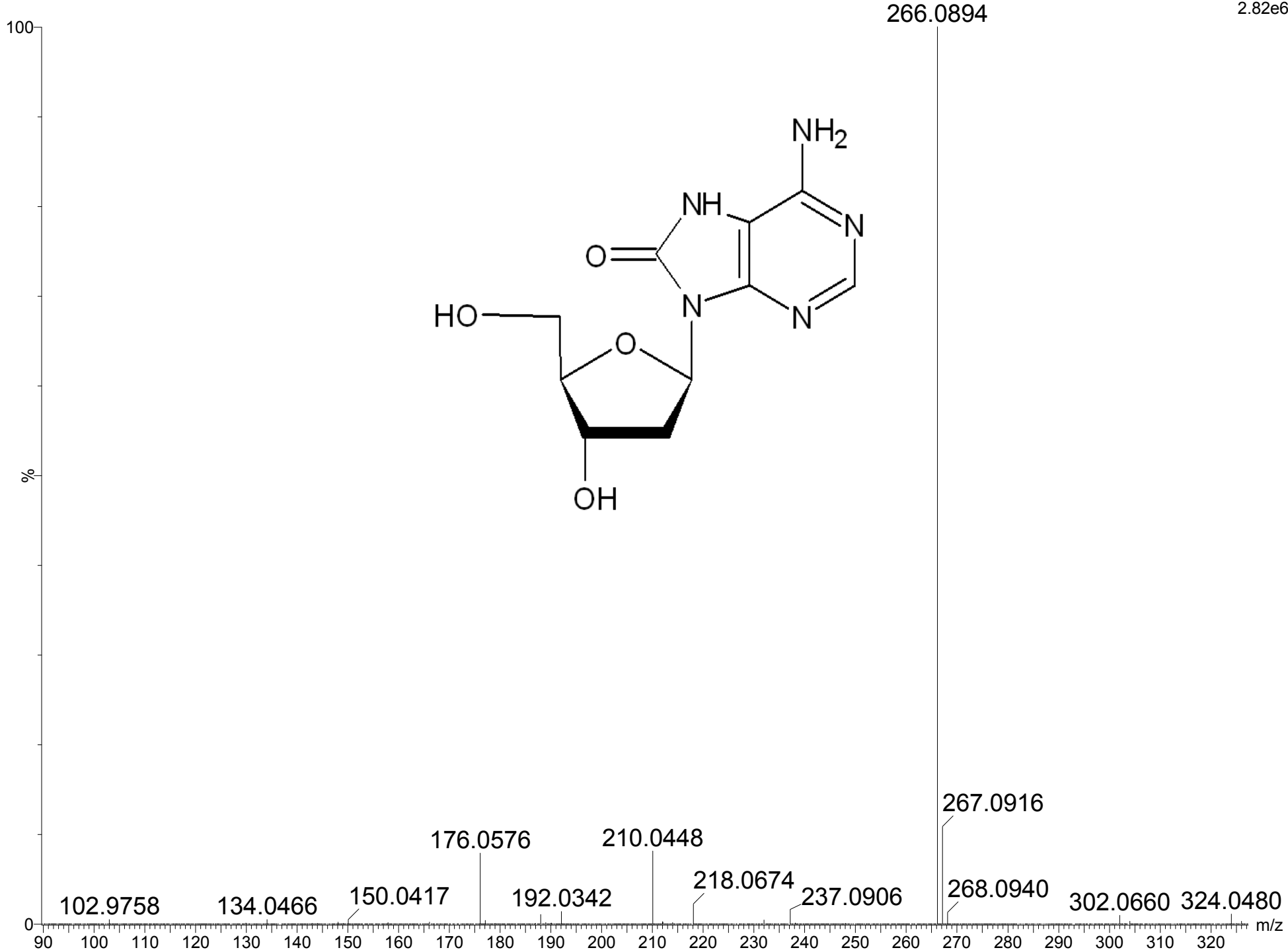

Supplement: Supplementary file 1 [file cells-14-01665-s001.zip › ESI MS spectra/8oxodAdo_esi_spec_neg.pdf]

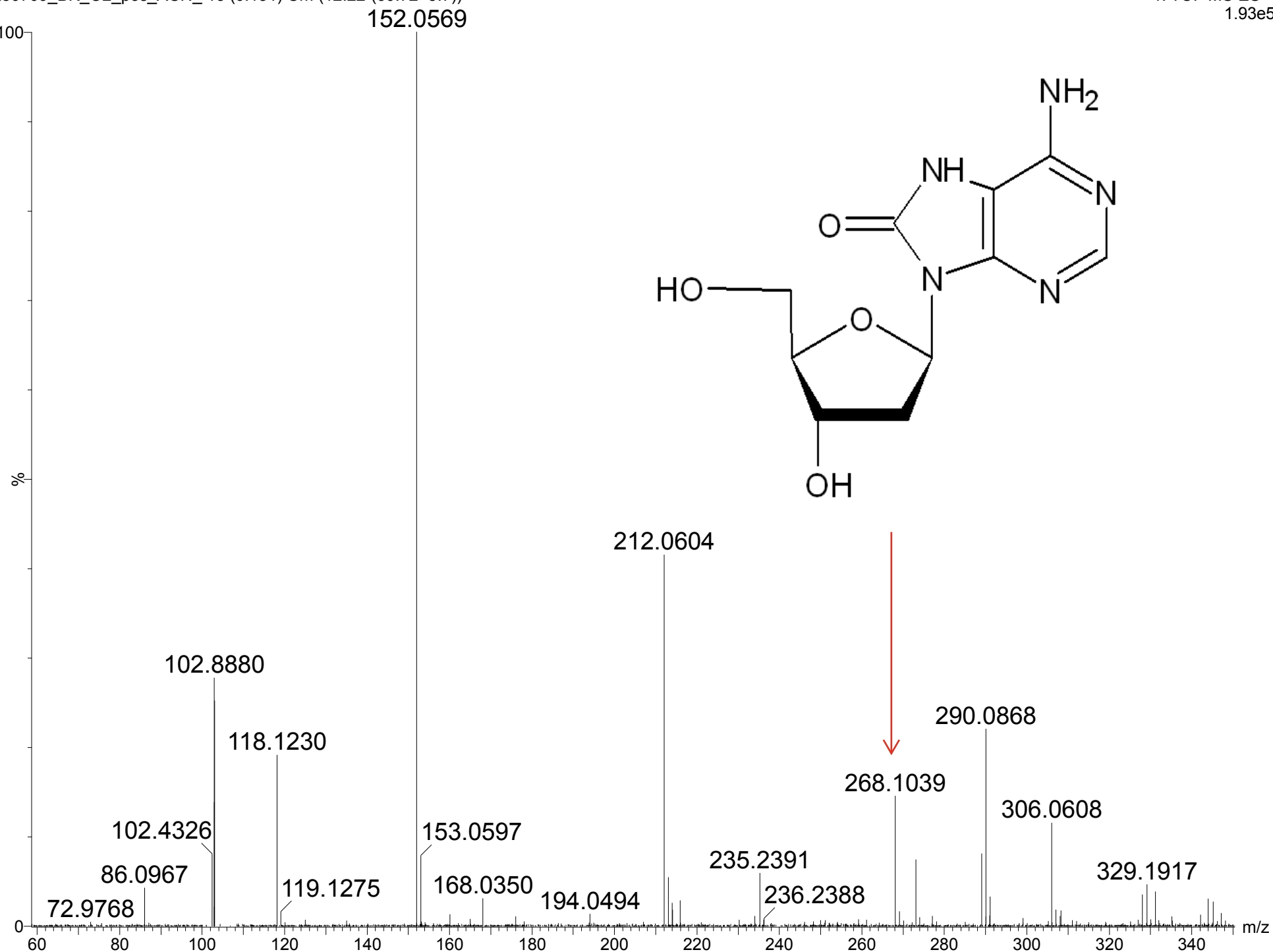

Supplement: Supplementary file 1 [file cells-14-01665-s001.zip › ESI MS spectra/8oxodAdo_esi_spec_pos.pdf]

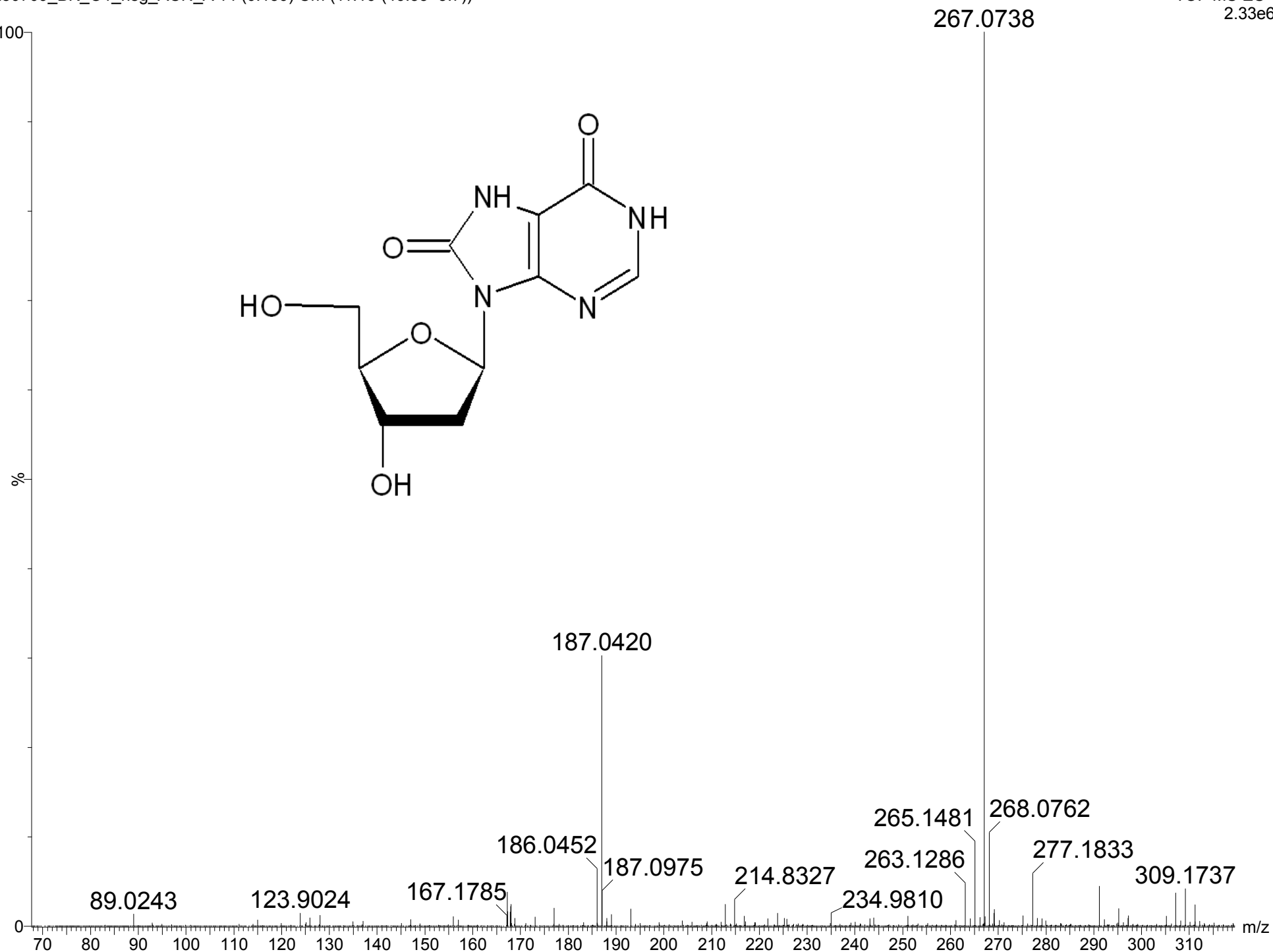

Supplement: Supplementary file 1 [file cells-14-01665-s001.zip › ESI MS spectra/8oxodIno_esi_spec_neg.pdf]

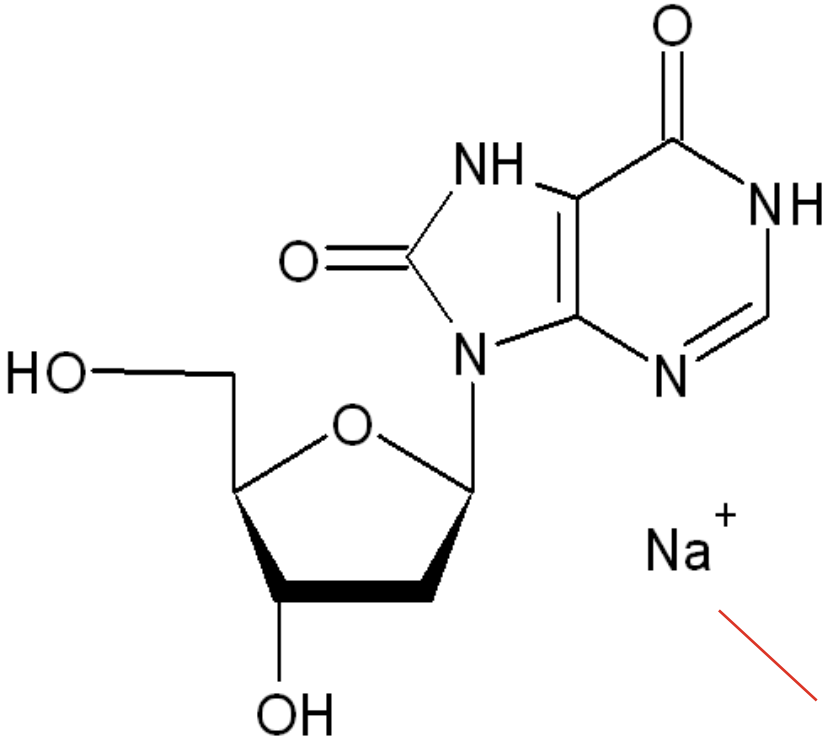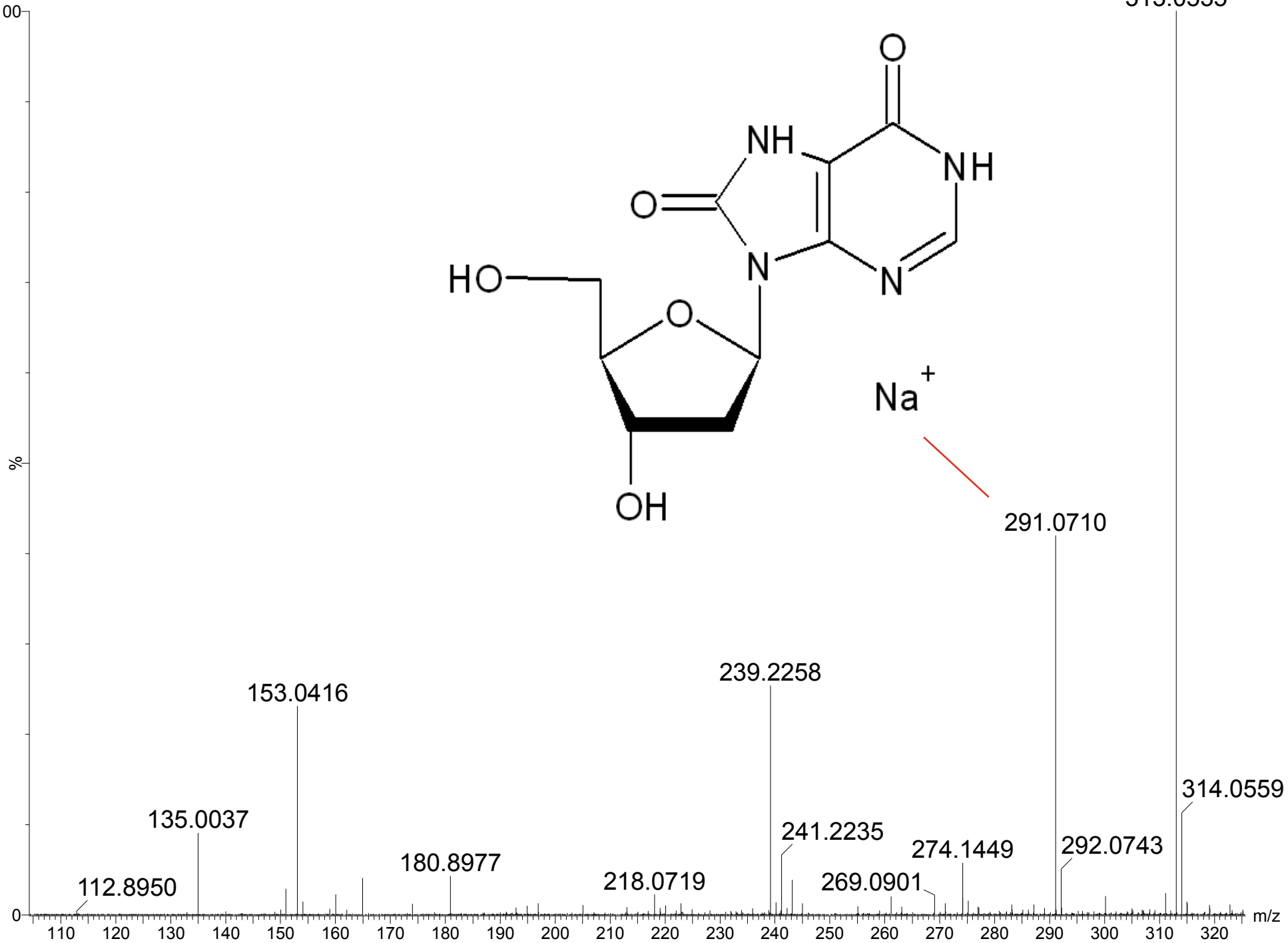

Supplement: Supplementary file 1 [file cells-14-01665-s001.zip › ESI MS spectra/8oxodIno_esi_spec_pos.pdf]

250710\_BK\_R2\_neg\_MSMS\_3 (0.151) Cm (3:4-9)

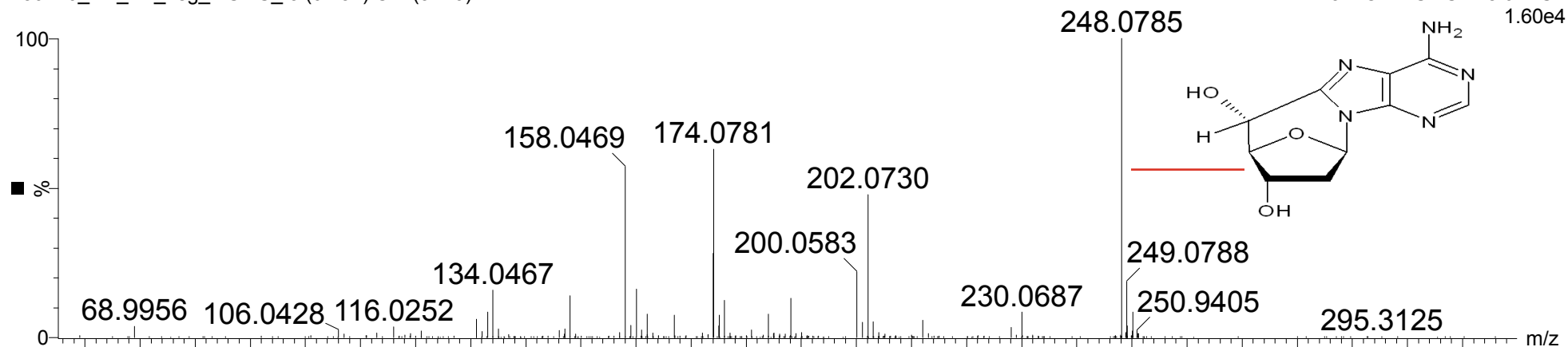

250710\_BK\_R2\_neg\_MSMS\_4 (0.176) Cm (4-11)

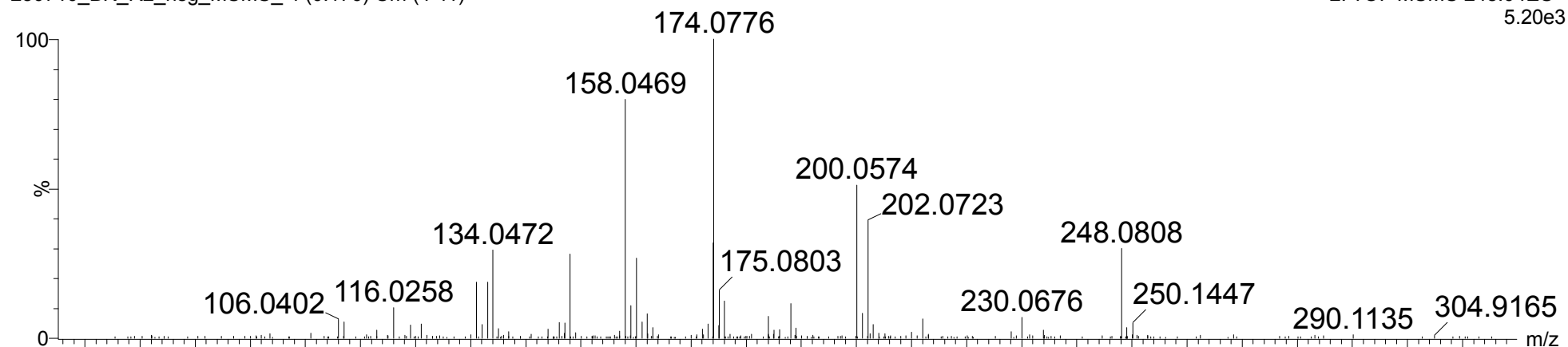

250710\_BK\_R2\_neg\_MSMS\_4 (0.168) Cm (4)

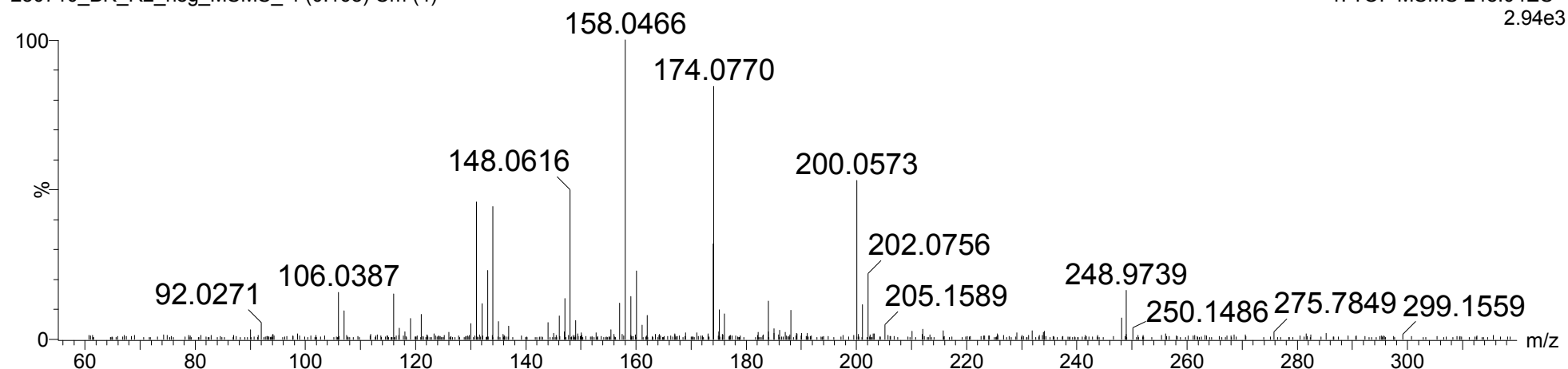

Supplement: Supplementary file 1 [file cells-14-01665-s001.zip › MS_MS spectra/(5R)cdAdo_MSMS_neg.pdf]

250710\_BK\_R2\_pos\_MSMS\_3 (0.151) Cm (3:4)

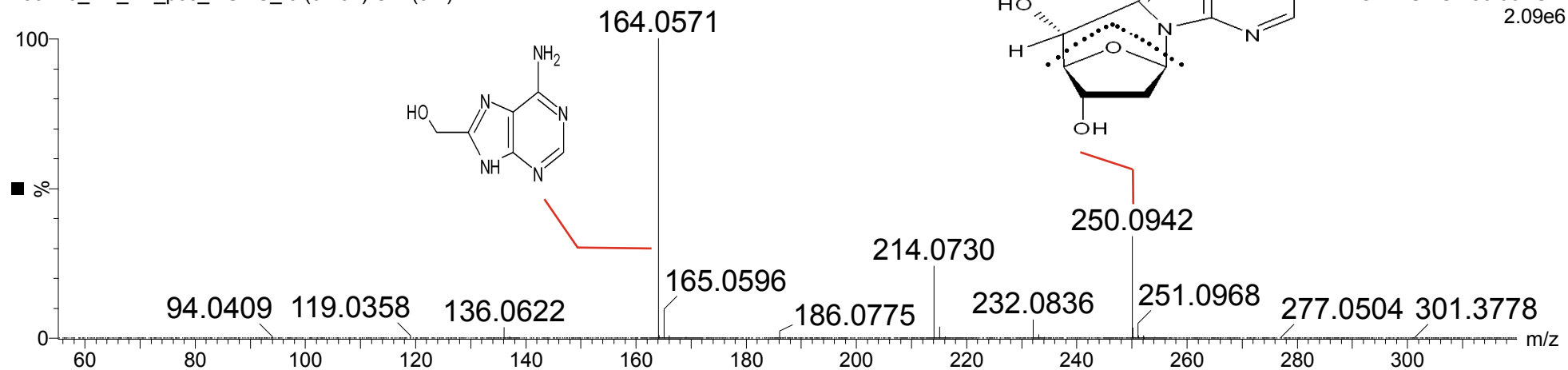

250710\_BK\_R2\_pos\_MSMS\_3 (0.176) Cm (3:4)

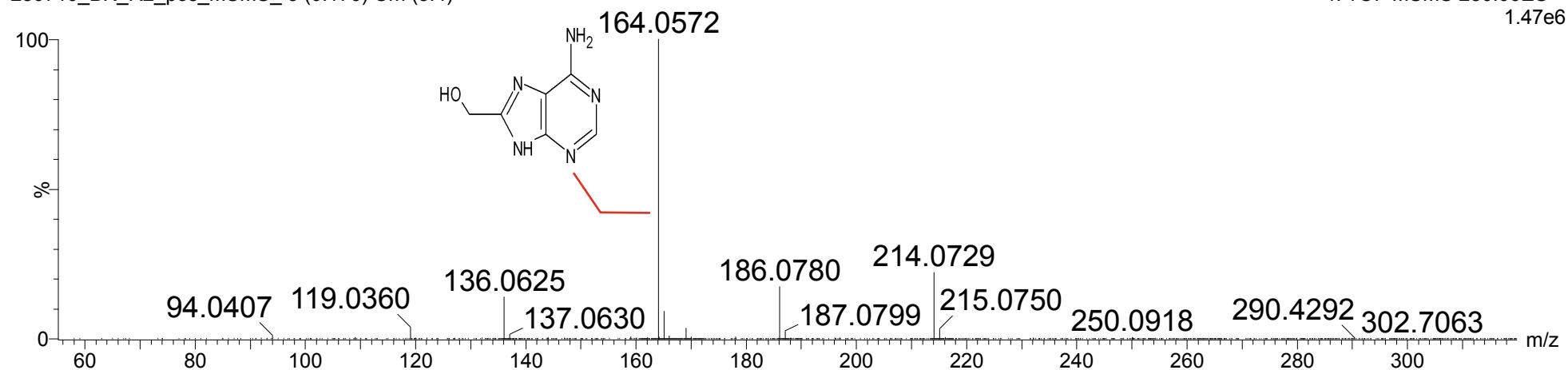

250710\_BK\_R2\_pos\_MSMS\_4 (0.230) Cm (4)

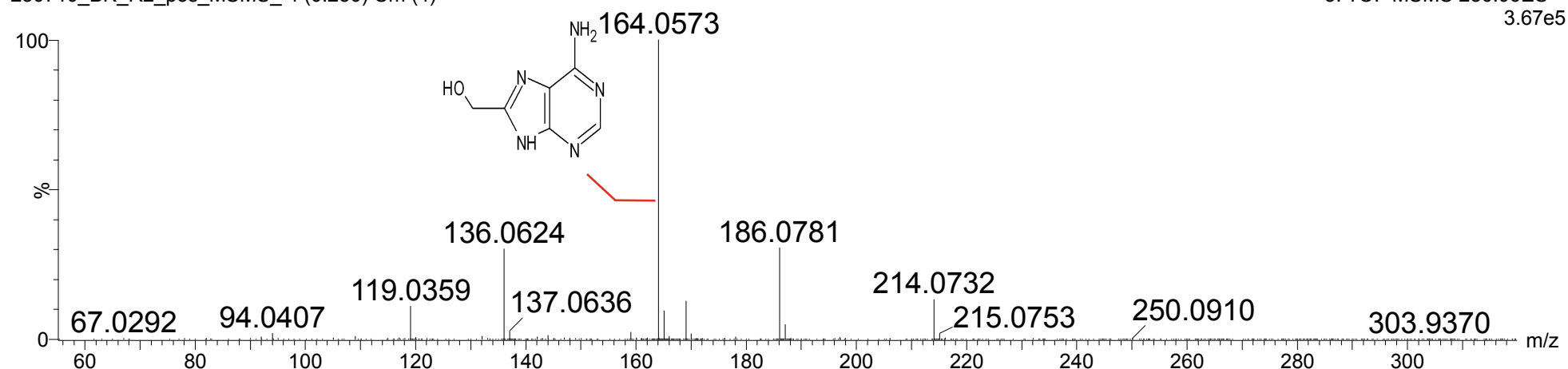

Supplement: Supplementary file 1 [file cells-14-01665-s001.zip › MS_MS spectra/(5R)cdAdo_MSMS_pos.pdf]

250710\_BK\_R1\_neg\_MSMS\_4 (0.185) Cm (3:5)

3: TOF MSMS 249.03ES-  
1.92e5

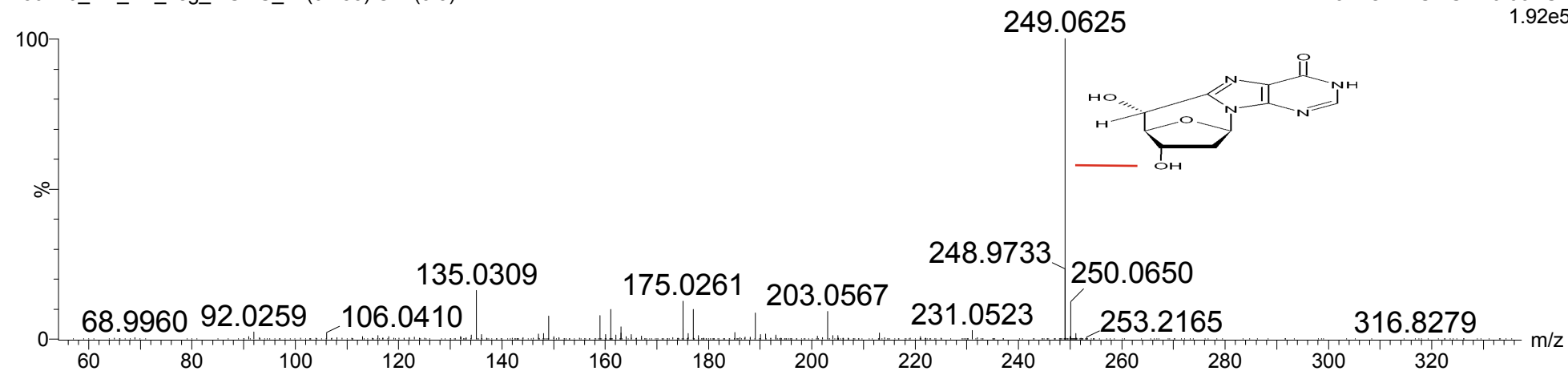

250710\_BK\_R1\_neg\_MSMS\_4 (0.176) Cm (3:5)

2: TOF MSMS 249.03ES-  
4.99e4

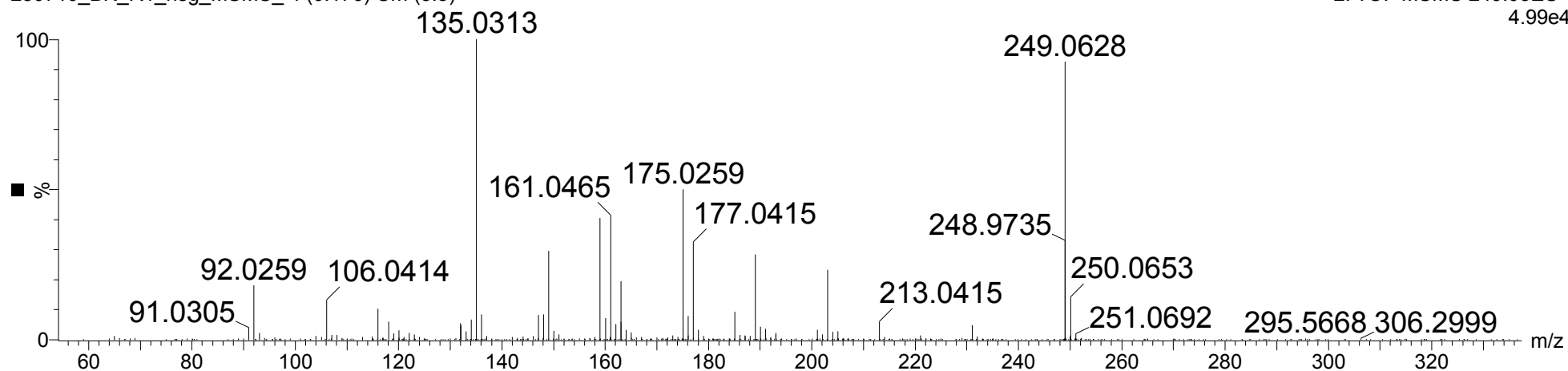

250710\_BK\_R1\_neg\_MSMS\_4 (0.168) Cm (3:5)

1: TOF MSMS 249.03ES-  
4.99e4

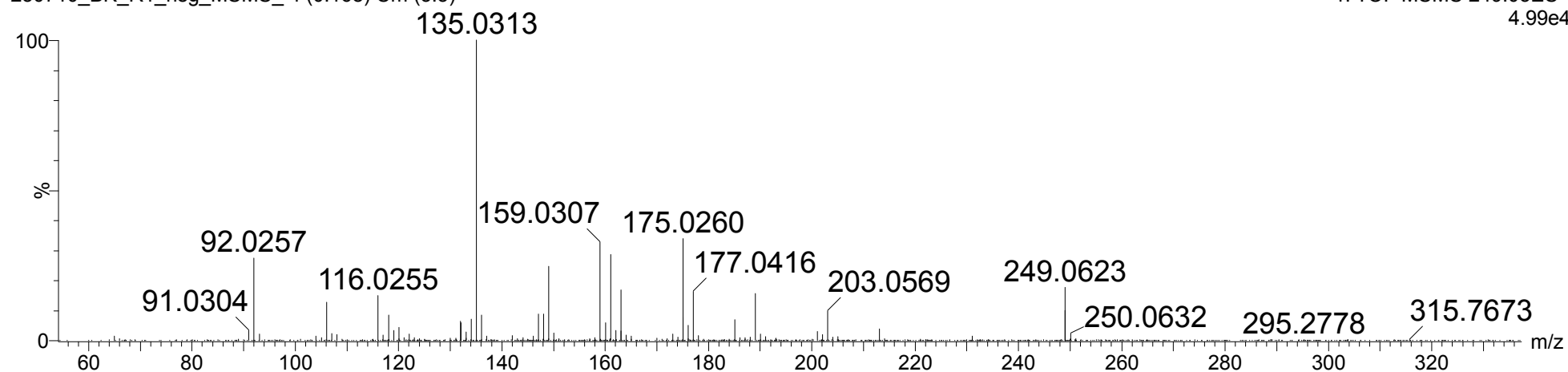

Supplement: Supplementary file 1 [file cells-14-01665-s001.zip › MS_MS spectra/(5R)cdIno_ MSMS_neg.pdf]

250710\_BK\_R1\_pos\_MSMS\_3 (0.151) Cm (3:5)

1: TOF MSMS 251.05ES+  
1.82e5

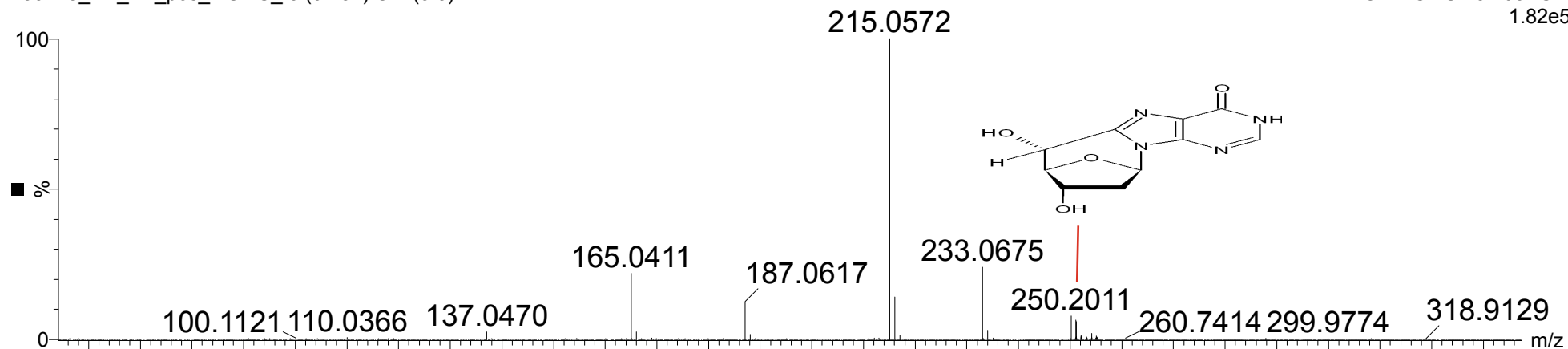

250710\_BK\_R1\_pos\_MSMS\_3 (0.176) Cm (2:4)

4: TOF MSMS 251.05ES+  
2.06e4

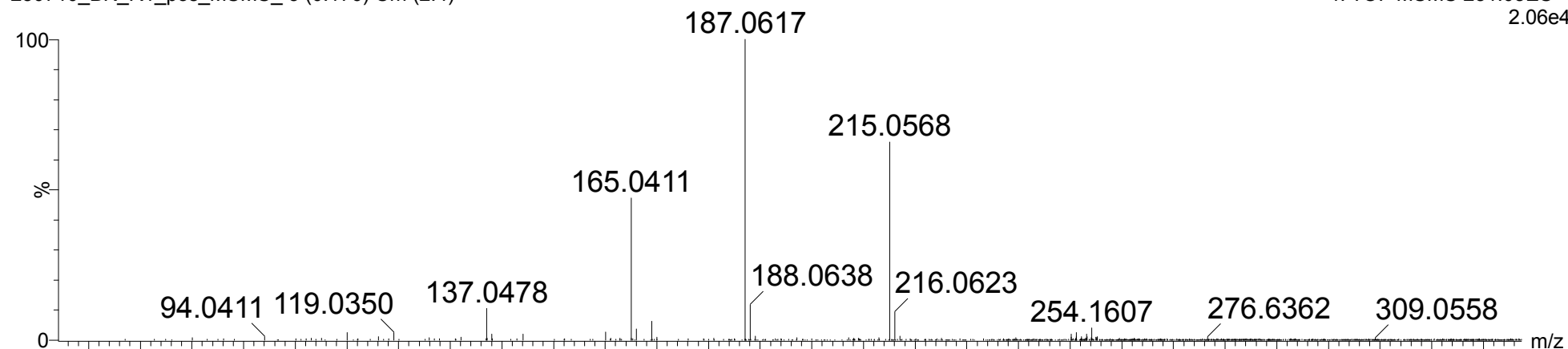

250710\_BK\_R1\_pos\_MSMS\_3 (0.168) Cm (3:4)

3: TOF MSMS 251.05ES+  
3.78e4

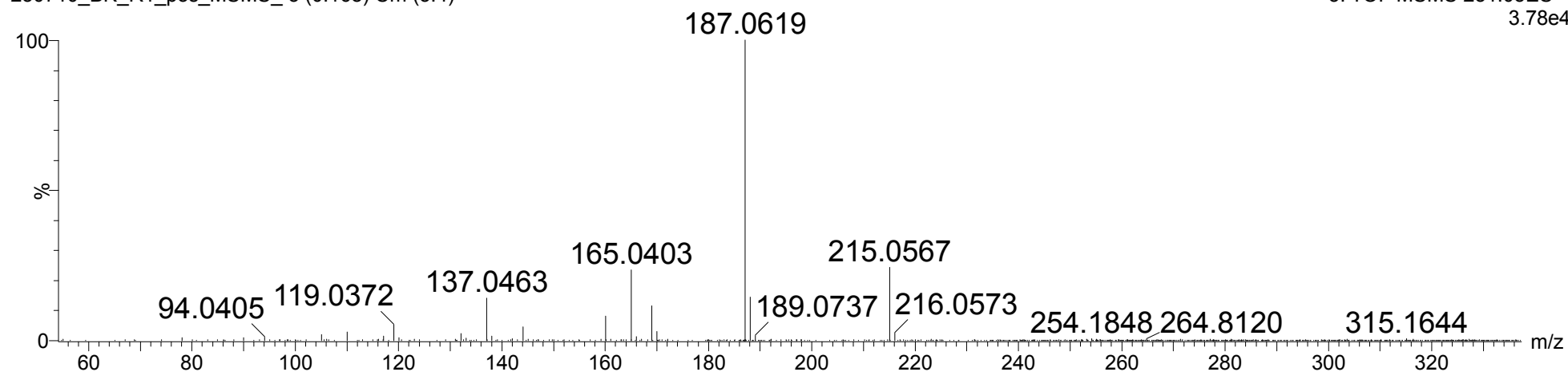

Supplement: Supplementary file 1 [file cells-14-01665-s001.zip › MS_MS spectra/(5R)cdIno_MSMS_pos.pdf]

250710\_BK\_S3\_pos\_MSMS\_3 (0.151) Cm (3:5)

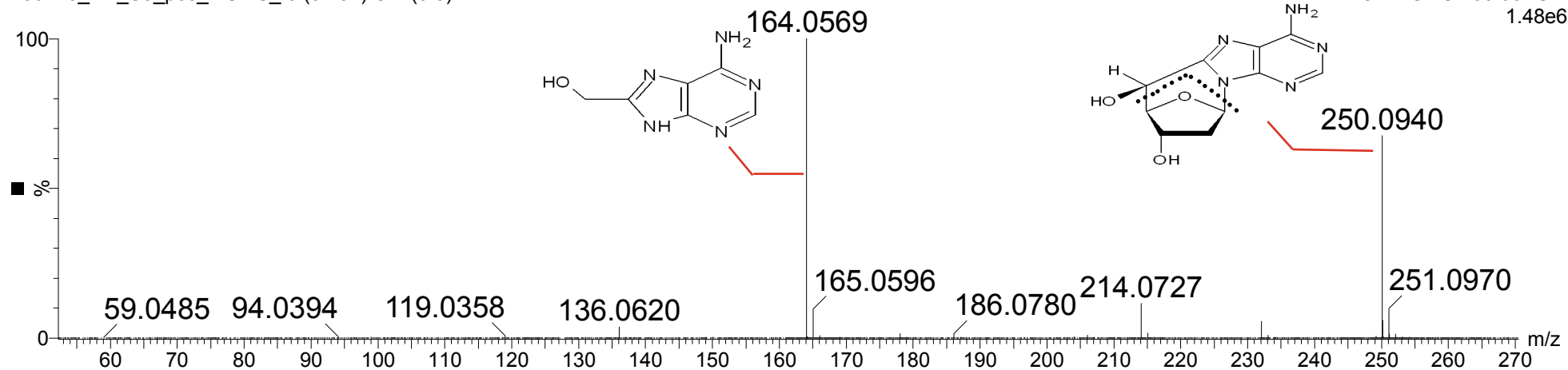

250710\_BK\_S3\_pos\_MSMS\_3 (0.176) Cm (3:4)

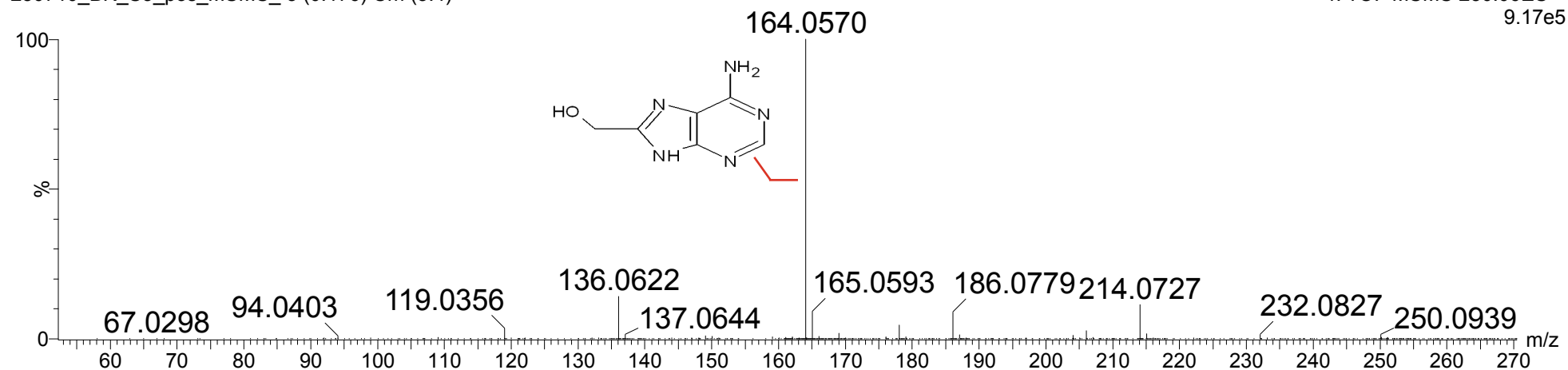

250710\_BK\_S3\_pos\_MSMS\_3 (0.168) Cm (3)

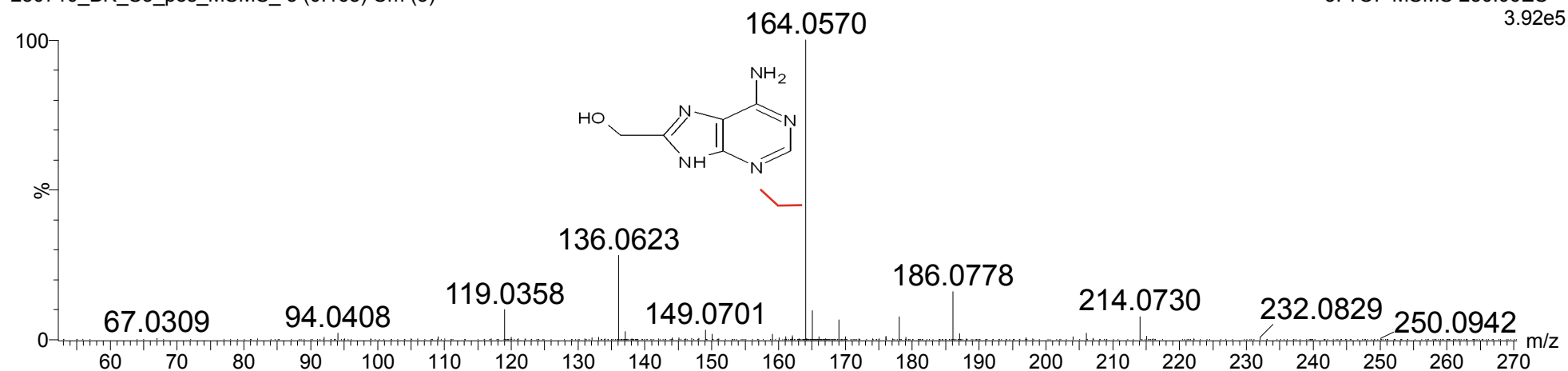

Supplement: Supplementary file 1 [file cells-14-01665-s001.zip › MS_MS spectra/(5S)cdAdo_MSMS_pos.pdf]

250710\_BK\_S2\_neg\_MSMS\_4 (0.185) Cm (4:5)

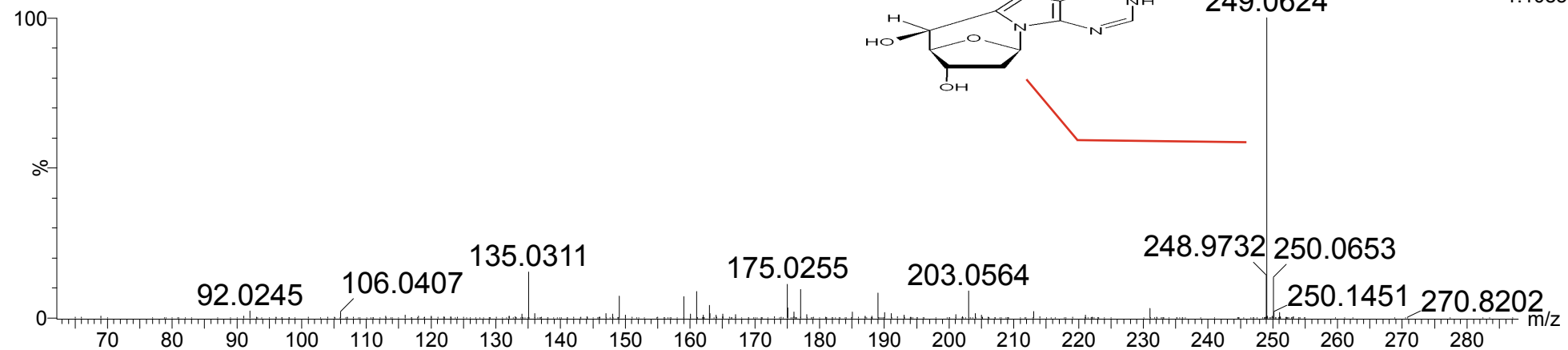

250710\_BK\_S2\_neg\_MSMS\_5 (0.230) Cm (4:5)

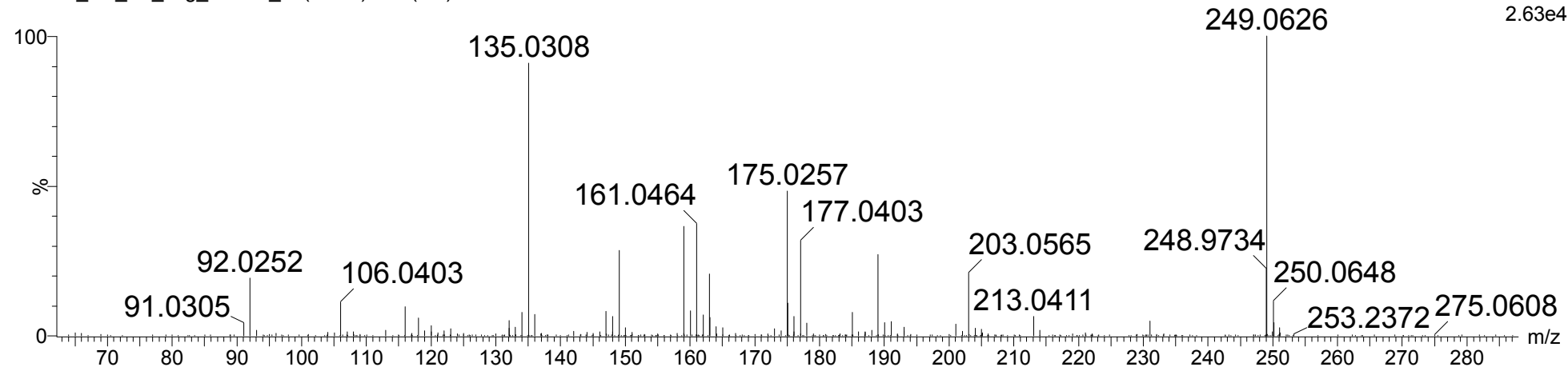

250710\_BK\_S2\_neg\_MSMS\_4 (0.168) Cm (4:6)

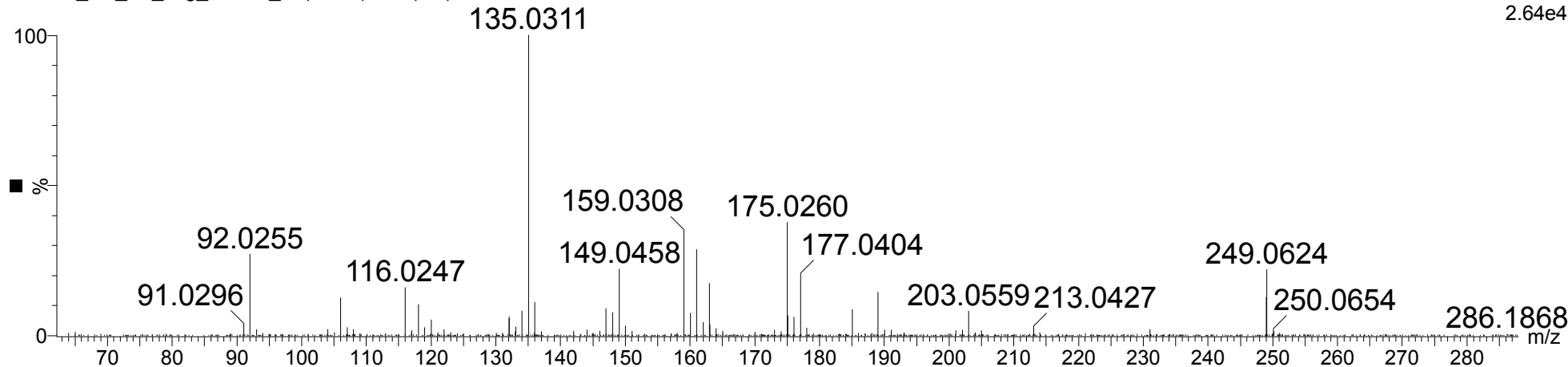

Supplement: Supplementary file 1 [file cells-14-01665-s001.zip › MS_MS spectra/(5S)cdIno_MSMS_neg.pdf]

250710\_BK\_S2\_pos\_MSMS\_3 (0.151) Cm (3:4)

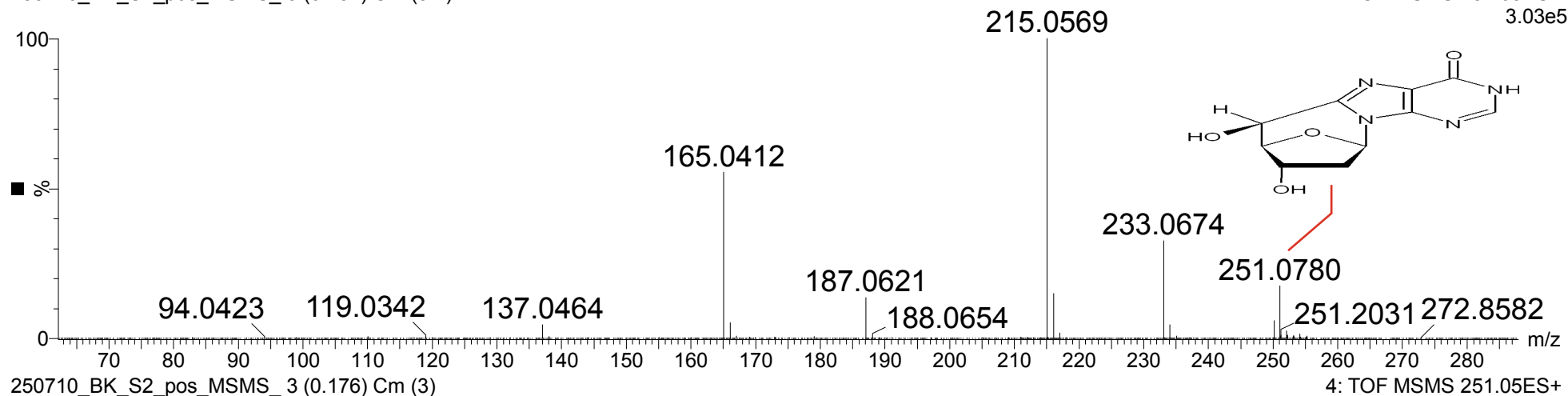

250710\_BK\_S2\_pos\_MSMS\_3 (0.176) Cm (3)

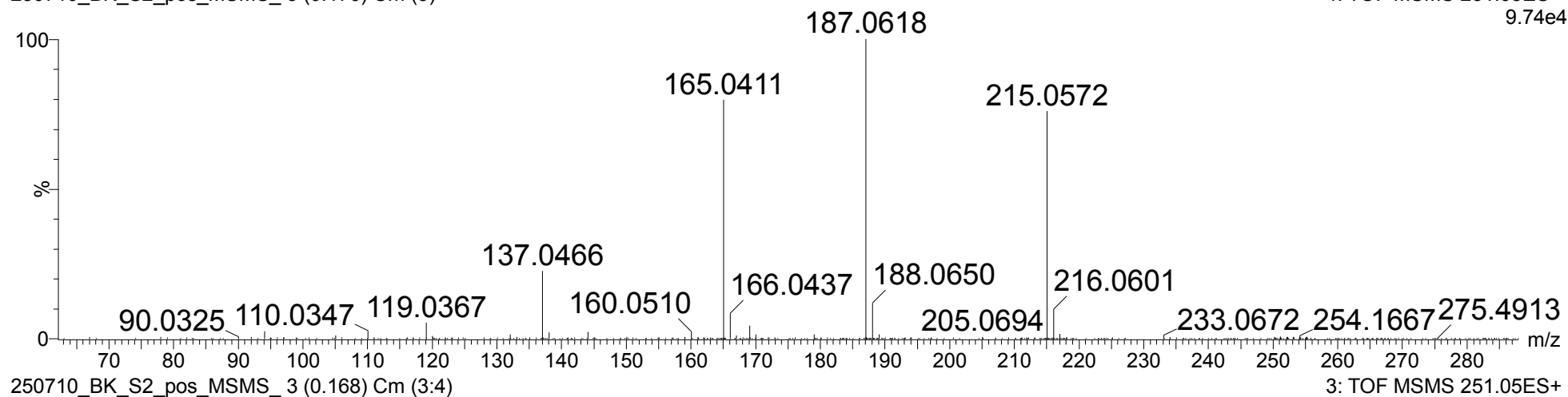

250710\_BK\_S2\_pos\_MSMS\_3 (0.168) Cm (3:4)

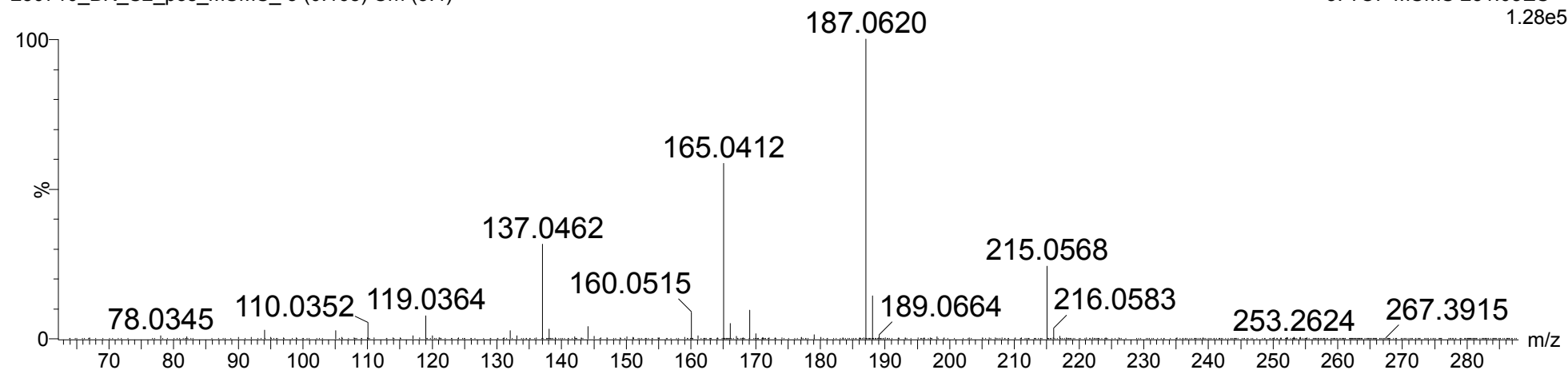

Supplement: Supplementary file 1 [file cells-14-01665-s001.zip › MS_MS spectra/(5S)cdIno_MSMS_pos.pdf]

250710\_BK\_S3\_neg\_MSMS\_3 (0.151) Cm (3:4)

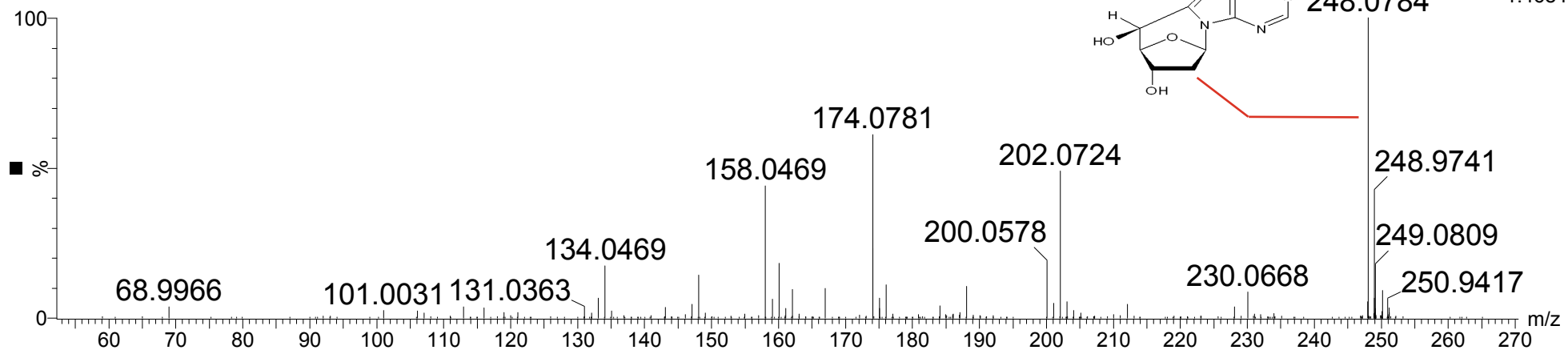

250710\_BK\_S3\_neg\_MSMS\_4 (0.176) Cm (3:4-14)

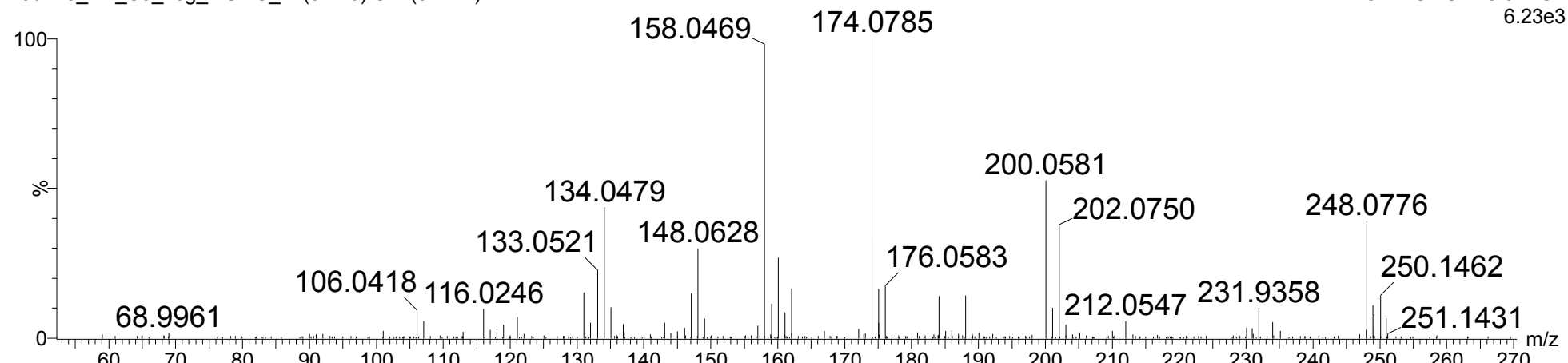

250710\_BK\_S3\_neg\_MSMS\_4 (0.168) Cm (3:5)

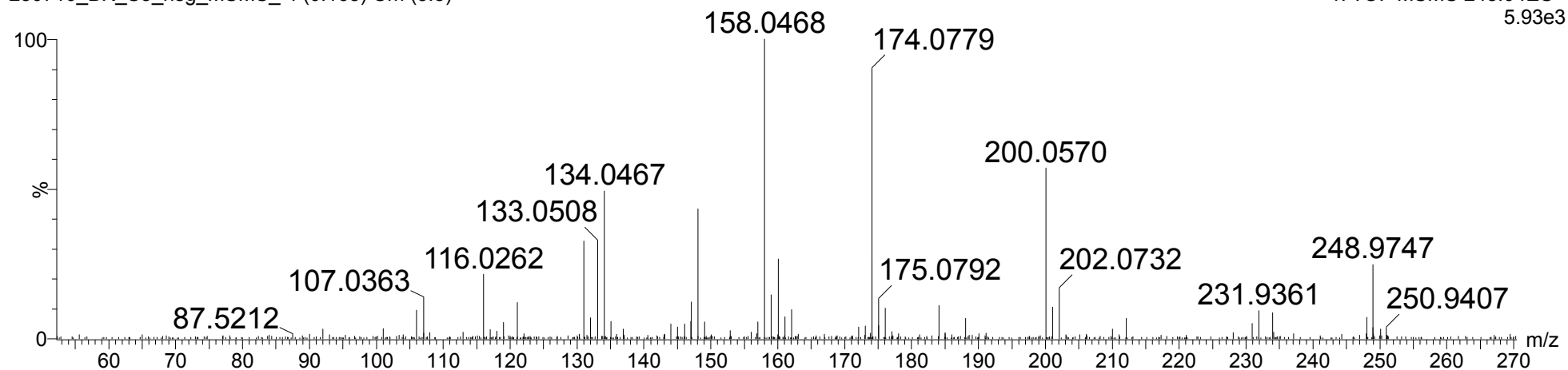

Supplement: Supplementary file 1 [file cells-14-01665-s001.zip › MS_MS spectra/(5S)dAdo_MSMS_neg.pdf]

250710\_BK\_O2\_neg\_MSMS\_3 (0.151) Cm (3:7)

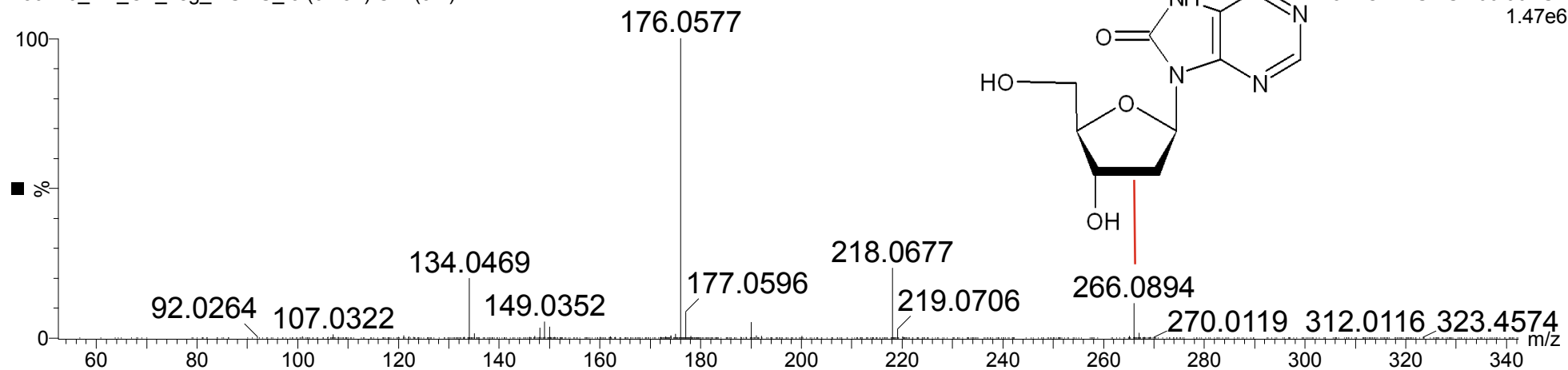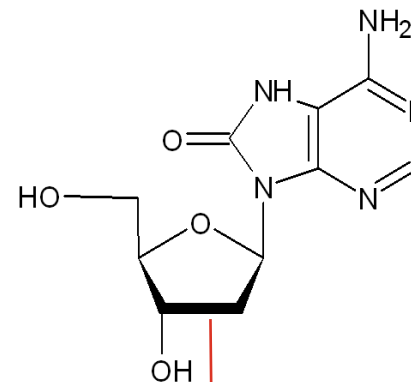

250710\_BK\_O2\_neg\_MSMS\_4 (0.176) Cm (4:5)

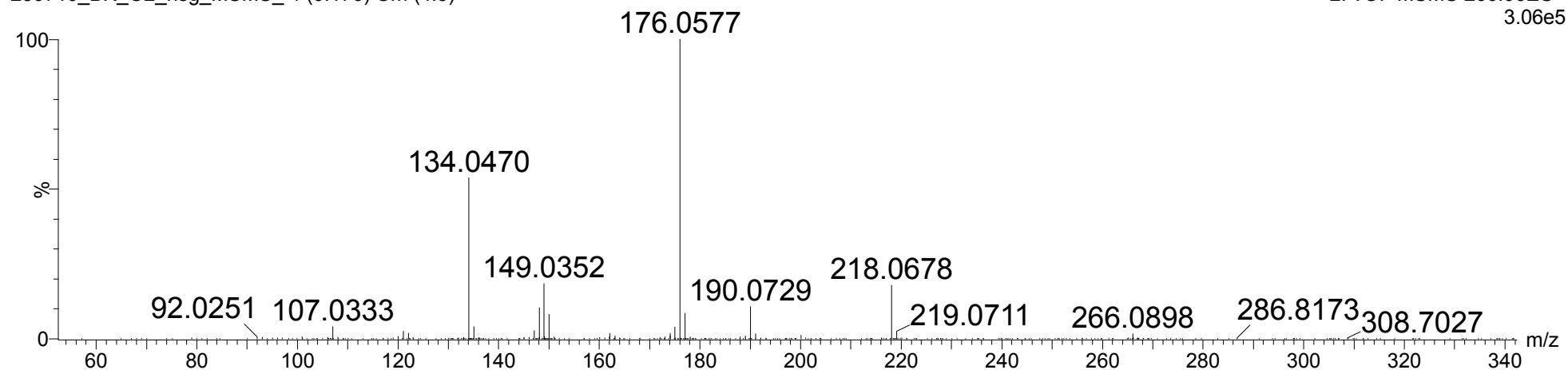

250710\_BK\_O2\_neg\_MSMS\_4 (0.168) Cm (4:6)

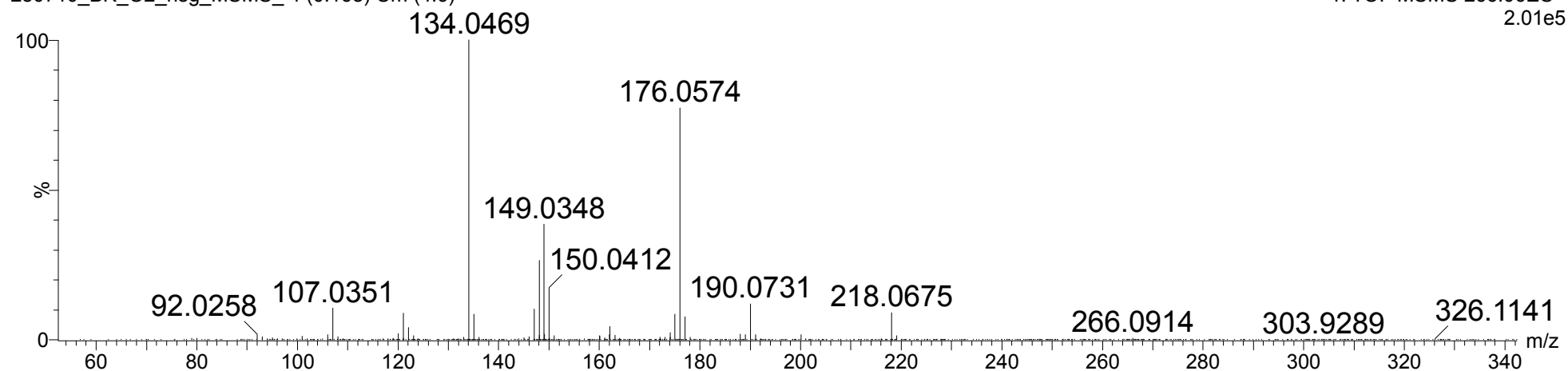

Supplement: Supplementary file 1 [file cells-14-01665-s001.zip › MS_MS spectra/8oxodAdo_MSMS_neg.pdf]

250710\_BK\_O2\_pos\_MSMS\_3 (0.151) Cm (3:7)

1: TOF MSMS 268.07ES+  
1.28e5

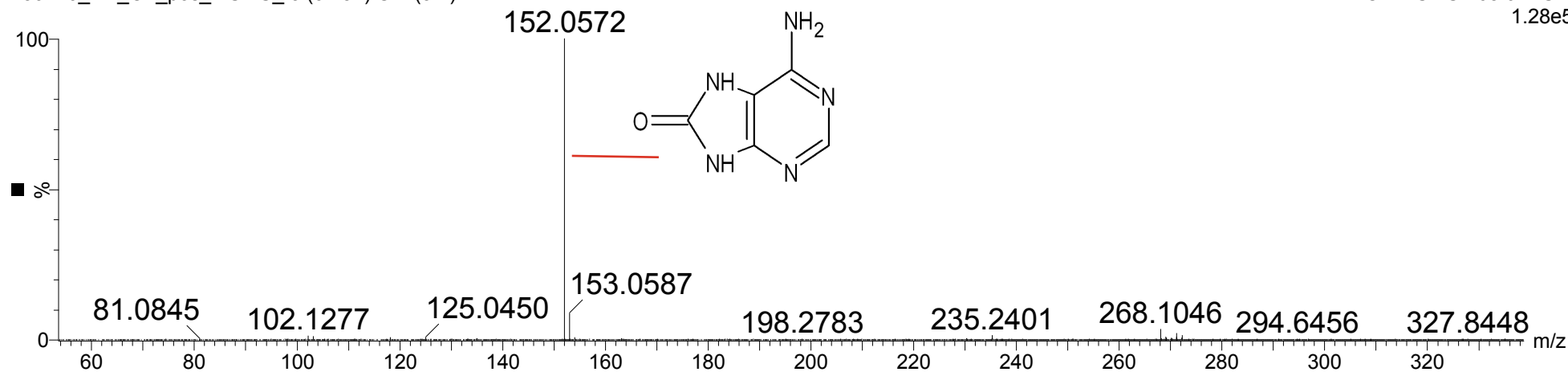

250710\_BK\_O2\_pos\_MSMS\_3 (0.176) Cm (3:6)

4: TOF MSMS 268.07ES+  
6.77e4

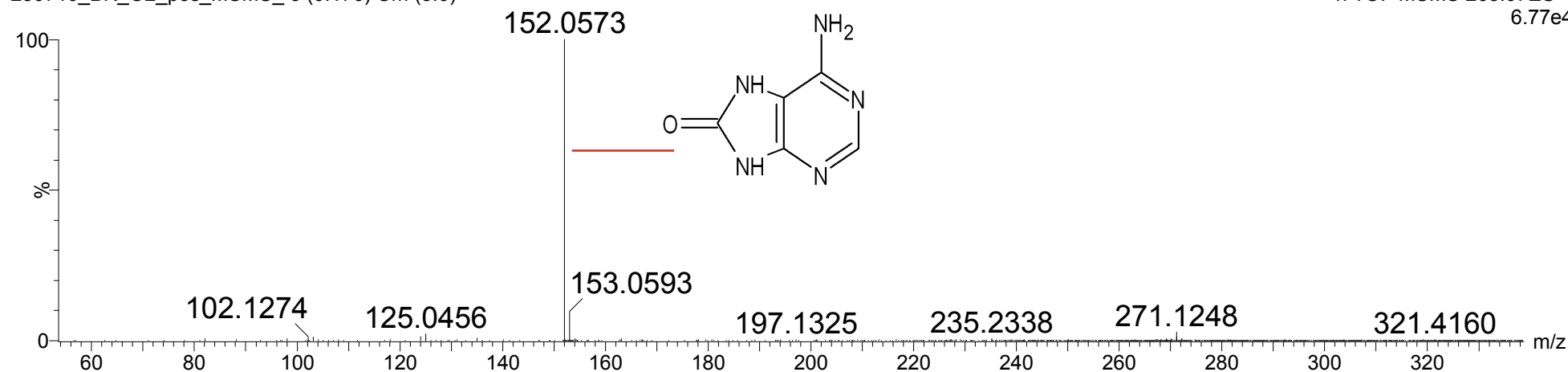

250710\_BK\_O2\_pos\_MSMS\_3 (0.168) Cm (3:5)

3: TOF MSMS 268.07ES+  
5.11e4

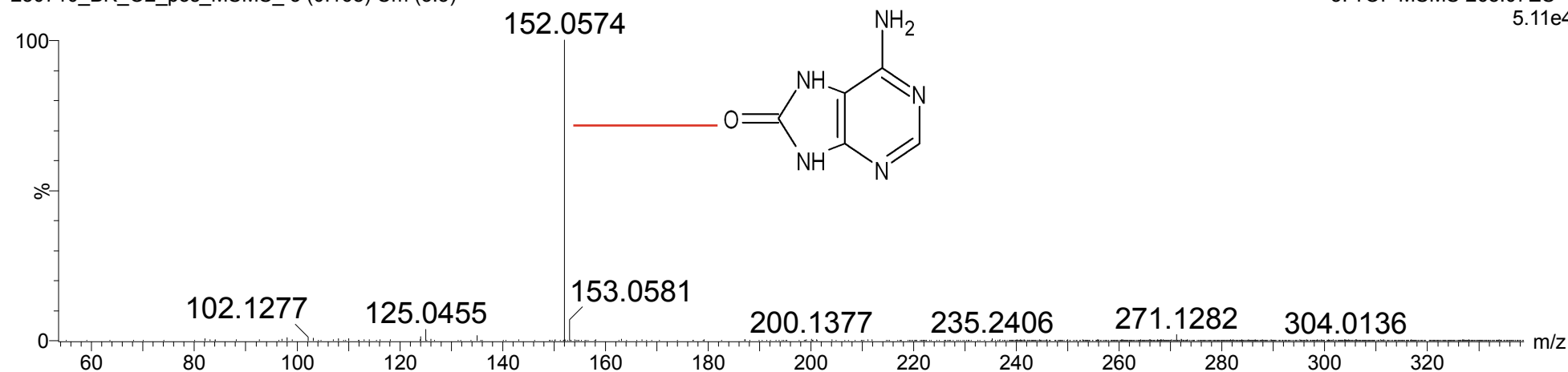

Supplement: Supplementary file 1 [file cells-14-01665-s001.zip › MS_MS spectra/8oxodAdo_MSMS_pos.pdf]

250710\_BK\_O1\_neg\_MSMS\_3 (0.151) Cm (2:4)

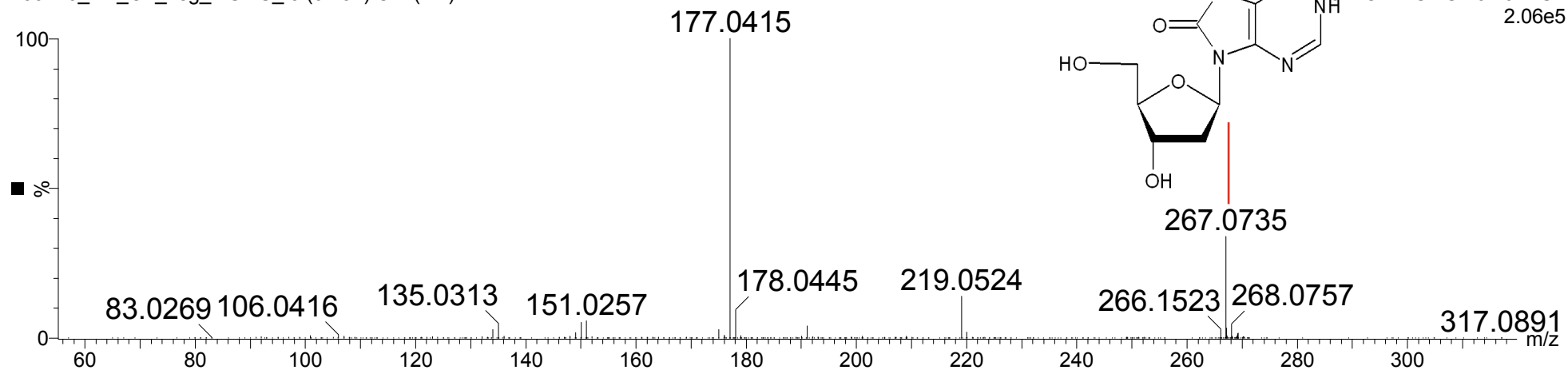

250710\_BK\_O1\_neg\_MSMS\_4 (0.176) Cm (3:4)

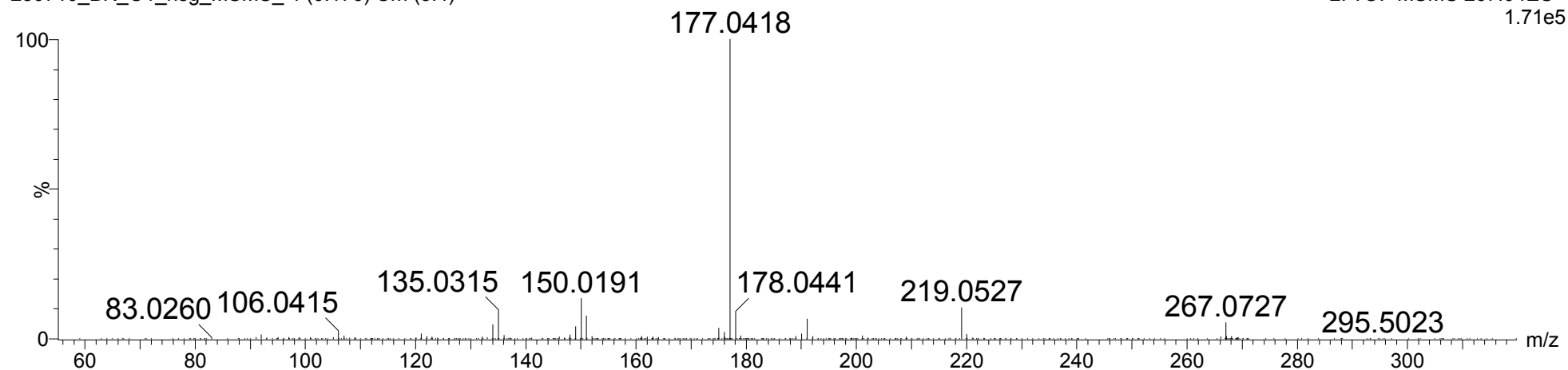

250710\_BK\_O1\_neg\_MSMS\_4 (0.168) Cm (3:4)

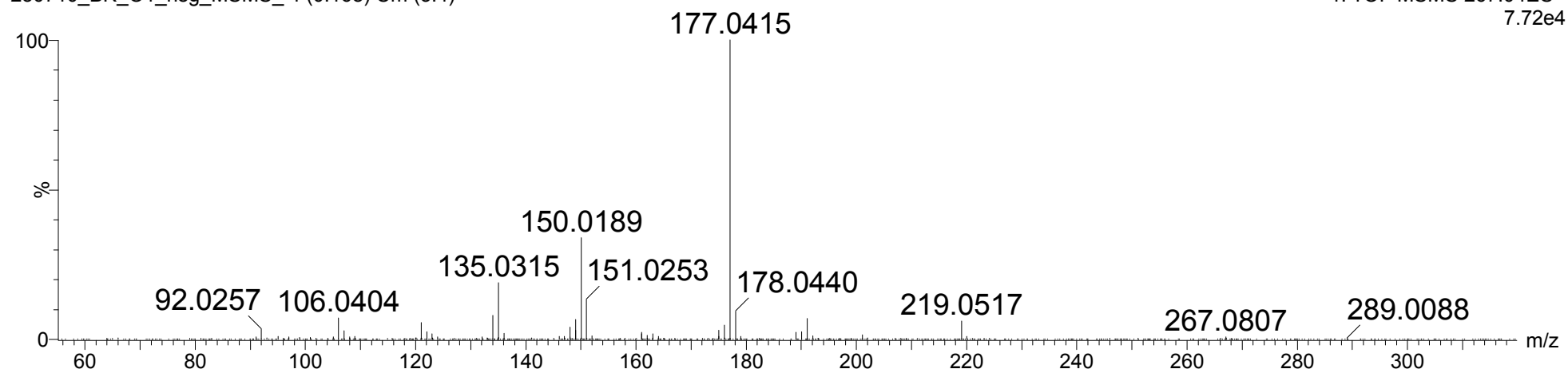

Supplement: Supplementary file 1 [file cells-14-01665-s001.zip › MS_MS spectra/8oxodIno_MSMS_neg.pdf]

250710\_BK\_O1\_pos\_MSMS\_3 (0.151) Cm (3:4)

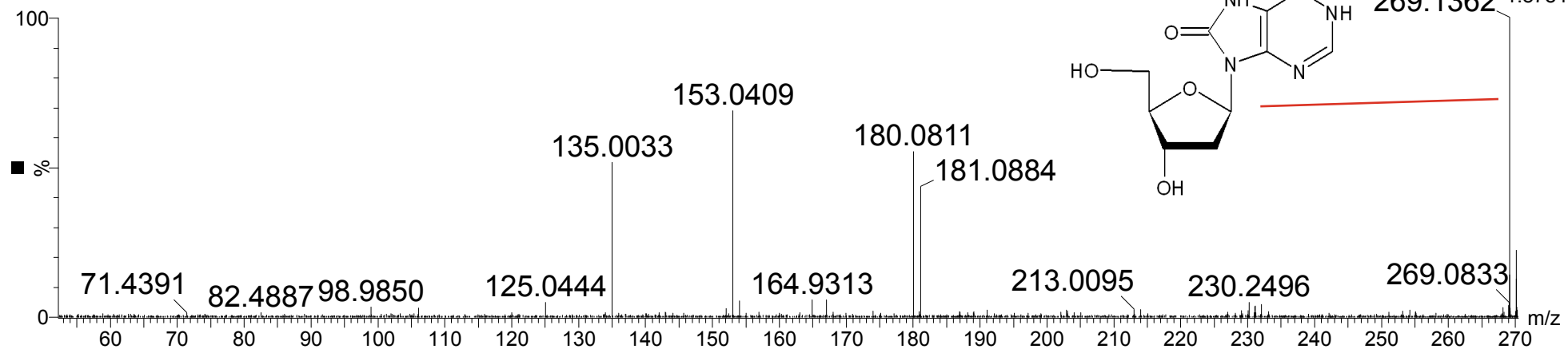

250710\_BK\_O1\_pos\_MSMS\_3 (0.176) Cm (2:3)

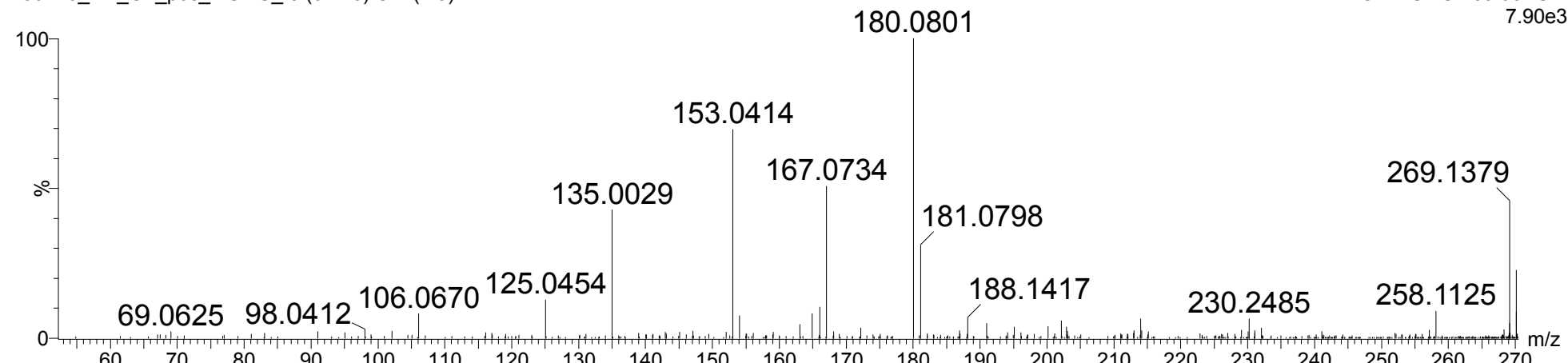

250710\_BK\_O1\_pos\_MSMS\_3 (0.168) Cm (3)

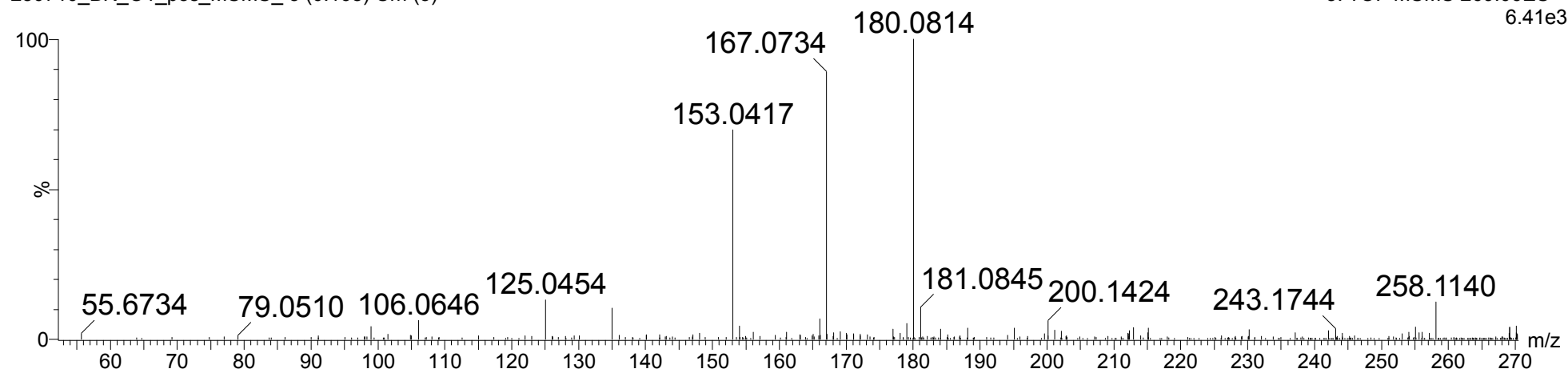

Supplement: Supplementary file 1 [file cells-14-01665-s001.zip › MS_MS spectra/8oxodIno_MSMS_pos.pdf]
